# Chameleon Swarm Algorithm Applied to Extract the Parameters of Photovoltaic Cells and Panels

**DOI:** 10.3390/biomimetics11100706

**Published:** 2026-10-03

**Authors:** Manoharan Madhiarasan, Petru A. Cotfas, Daniel T. Cotfas

**Affiliations:** 1 Independent Researcher, No. 14, Uzhaippaali Street, Annai Anjugam Nagar, Ayapakkam, Chennai 600077, Tamil Nadu, India; 2 Department of Electronics and Computers, Faculty of Electrical Engineering and Computer Science, Transilvania University of Brașov, 500036 Brasov, Romania; pcotfas@unitbv.ro

**Keywords:** photovoltaic cells and modules, parameters, metaheuristic algorithms, CSA algorithm

## Abstract

Forecasting the energy output of photovoltaic modules is an important consideration for prosumers and investors. The forecast accuracy depends on the precise determination of key external and internal parameters. This study proposes applying the metaheuristic Chameleon Swarm Algorithm (CSA) to extract the parameters for two photovoltaic cells, RTC France and aSi:H amorphous silicon, and one photovoltaic module, PWP 201. The CSA is applied to the most widely used models, which derive from the equivalent circuits for the photovoltaic cells and modules, namely with one, two, and three diodes. Six statistical metrics are used to verify and validate the performance of the CSA. The study also reports the following metrics: mean, standard deviation, minimum, maximum and interquartile range to evaluate the reliability and robustness of the algorithm. It is compared with several algorithms from 2023 to 2025 and some very good algorithms from outside of this interval. Computing time is also analyzed depending on the photovoltaic cells and module, the three models, and the number of iterations. The results for the main statistical metrics, root mean square error (RMSE), and computing time prove that the CSA is one of the most effective and reliable. The obtained RMSE is the best in some cases or equal, and the computing time is very low, thus certifying that the CSA is competitive against, and in several cases improves upon, previously reported best-known algorithms’ results.

## 1. Introduction

Addressing climate change and ensuring the sustainability and viability of the energy sector are crucial issues. The fossil fuels used to generate energy contribute to the greenhouse effect, pollution, and environmental damage, and they are limited resources. The increasing demand for sustainable and affordable energy solutions has therefore sped up the shift towards renewable energy solutions.

Renewable energy, such as solar, wind, hydro, geothermal, biomass, and waves, is crucial for this global energy transition [1]. Solar energy represents a huge advantage as it is inexhaustible, abundant, and globally distributed. Photovoltaic panels (PV) convert this renewable energy in electricity.

Considering both energy demands and the need for sustainability, the national policies and economies of countries must adapt to the new paradigms. In this context, the European Union has adopted a series of directives where solar energy is considered one of the central pillars for its climate policy. EU solar energy strategy proposed as part of REPowerEU has some initiatives, such as the European Solar Rooftops Initiative, the EU large-scale skills partnership for renewable energy, and the EU Solar PV Industry Alliance, which aim to have a cumulative generation of 600 GW in 2030 [2]. The road map for installed PV capacity shows a remarkable trajectory: capacity tripled from 2018 to 2023, is projected to constitute 80% of the total renewable capacity installed, and by the end of 2030 it will be the main renewable source [3].

Accurate parameter extraction of solar cells is essential for performance evaluation, modeling, and optimization of photovoltaic panels. A huge issue with photovoltaic systems is their variability in energy generation due to the variation in solar radiation. To mitigate this issue, it is crucial to develop strong forecasts for the energy generated by PV systems, for which accurate PV parameter extraction can represent a foundational requirement.

The fundamental parameters of a photovoltaic cell are: the photocurrent generated (*I*_*p*ℎ_), reverse saturation current (*I*_o_), diode ideality factor (*n*), parasite resistance series (*Rs*) and shunt (*Rsh*) [4]. The current-voltage characteristic and the mathematical models that result from the PV equivalent circuits are the tools used to obtain the parameters. The accuracy of the parameter extraction process is challenging due to the nonlinear and multimodal nature of the photovoltaic cell’s governing equations. The methods used to determine the PV parameters are analytical, numerical techniques and metaheuristic algorithms [5]. The first two methods, despite finding solutions rapidly, suffer from limitations, such as falling into local optima and sensitivity to initial guesses [6]. To overcome these challenges, metaheuristic optimization algorithms have recently gained increasing attention [7,8]. They are able to handle nonlinear optimization issues and extract the PV parameter with high accuracy. When evaluating novel metaheuristics against conventional methods and existing meta-heuristics, devising novel metaheuristics to solve optimization issues requires a systematic framework. Exploration refers to the ability of algorithms to broadly examine various regions of the search space, which is crucial for mitigating local optima traps and escaping stagnation. Exploitation is the capacity to find potential solutions locally across all significant domains and improve the problem-solving level. There are several classifications for metaheuristic algorithms used for PV parameter extraction. For instance, one taxonomy categorizes the algorithms into seven classes, such as swarm, physics, maths, chemical, evolutionary, human and plant-based [9].

**Figure 1:**
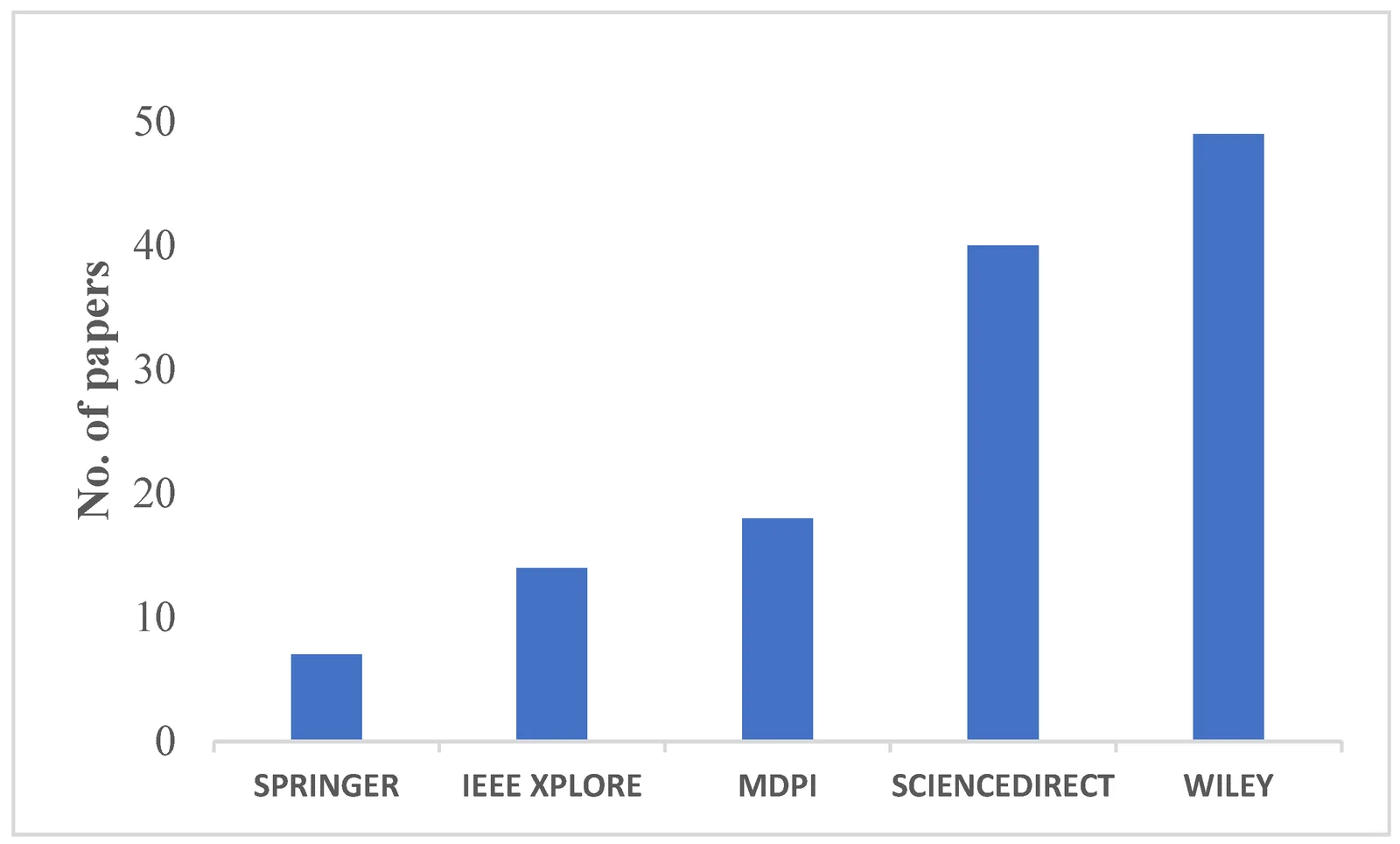
Number of papers published in five databases.

The determination of photovoltaic parameters began in the 1960s [10], with numerous methods being developed and presented in research papers, the subject still being in the attention of researchers, especially after the emergence and development of metaheuristic algorithms. This is easily observed by analyzing the papers presenting research on this topic from 2023 to 2025. The number of papers published, which is consistent, containing five very well-known studies, Springer, MDPI, IEEE Xplore, ScienceDirect and Wiley, in the years 2023–2025, is presented in Figure 1. More than 80% of these papers focus on metaheuristic algorithms, underscoring the importance of this topic and the “fight” to minimize the RMSE in parameter extraction, but also to reduce the computational time.

For the reasons mentioned above, this paper investigates the application of the Chameleon Swarm Algorithm (CSA) metaheuristic algorithm for photovoltaic cells and panels parameter extraction. Specifically, it evaluates the performance of CSA in terms of accuracy, convergence speed, and robustness in comparison with other metaheuristic algorithms.

The novelty of the contribution lies in a systematic and statistically supported evaluation of the CSA for photovoltaic parameter identification across three photovoltaic devices and three equivalent-circuit representations. Specifically, it emphasizes the following contributions:

1. A unified CSA-based parameter extraction framework is evaluated for the RTC France silicon cell, the aSi:H amorphous silicon cell, and the PWP201 photovoltaic module using single-diode, double-diode, and triple-diode models.

2. The study includes the aSi:H dataset, which broadens the assessment beyond the commonly used RTC France cell and PWP201 module benchmarks.

3. Each model’s different iteration configuration is executed 20 times with distinct random seeds rather than being represented by a single optimization run.

4. To validate the accuracy, reproducibility, stability, and robustness of CSA in extracting PV parameters, this study analyzes the RMSE, mean, standard deviation, min–max range, interquartile range (IQR), and coefficient of variation.

5. The results suggest that CSA is competitive against, and in several cases improves upon, previously reported best-known results.

The rest of the work is structured as follows: Section 2 reviews several algorithms developed and applied between 2023 and 2025, Section 3 details the CSA description, the equations used for PV parameter extraction and the flow chart, and Section 4 presents the equations for all three diode modes and for the photovoltaic module. Section 5 outlines the applied statistical metrics, evaluates the results of the CSA, and provides a comparative discussion. Section 6 analyzes the computational time, and Section 7 concludes the paper and outlines future work.

## 2. Related Works

A brief selection of key studies is highlighted from the over 100 references presented in Figure 1. The focus will be placed on the papers that discuss the extraction of PV parameters with very good results in terms of accuracy and time duration for extraction, and they will be in chronological order by year. Most of the authors use only the SDM and DDM models for the extraction of the PV parameters.

Beskirli and Dag proposed extracting the parameters of the photovoltaic cells and panels using the Improved Carnivorous Plant Algorithm (I-CPA) [9]. They used the one-diode (SDM) and two-diode (DDM) models for the RTC France photovoltaic cell and three modules: Photowatt-PWP201 (PWP), STM6-40/36 and KC200GT. The improved version of the CPA prevents the algorithm from becoming trapped in local minima, thereby enhancing the accuracy of parameter extraction. Comparison with CPA, Particle Swarm Optimization (PSO), Differential Evolution Algorithm (DE), Skill Optimization Algorithm (SOA) [11] and Coati Optimization Algorithm (COA) [12] shows that I-CPA outperforms them in terms of RMSE.

Belabbes et al. improved the snake optimization metaheuristic algorithm to enhance parameter extraction accuracy. The improvements ensure that there is always food and the weather is hot. These can be achieved if the limits for food quantity and temperature are changed [13]. Only the SDM and DDM modes were used for three photovoltaic cells, RTC, aSi and commercial mSi. The ISOA algorithm increases, for example, the accuracy by 0.1% for SDM and 2.8% for DDM, in the case of aSi. A thorough analysis is done of variations in the populations and the iteration number to reduce the calculation time. Computing time is lower than other competitors, but remains high, around 20s.

The hybrid successive discretization algorithm (HSDA) is used to extract the parameters for RTC and aSi photovoltaic cells and for four panels among PWP201, using all three models, SDM, DDM and TDM. Two statistical metrics are used for comparison: RMSE and MAE. The TDM model showed improved performance in comparison with the other two models in almost all cases. In the case of aSi, the improvements reach 89% and 84% compared to the SDM and DDM models, respectively [14].

Lai et al. introduce the Improved Artificial Protozoa Optimizer algorithm (IAPO), which offers an excellent balance between exploration and exploitation, to extract the parameters of the RTC photovoltaic cell using the SDM and DDM models. The RMSE is the main statistical test to validate and compare the results of IAPO with several other algorithms from the specialized literature. An analysis of RMSE variation is also made as a function of the iteration number. This number varies from 1000 to 10,000. It can be observed that the IPOA obtained the lowest RMSE after 2000 iterations for the SDM model, while the APO did so after 4000 iterations, respectively. In the DDM model, IAPO converges after 2000 iterations, while APO converges after 10,000 iterations [15].

Coati improved snow ablation optimization algorithm (CSAO) is used to extract the parameters of the PV cell, RTC France cell, and three panels, PWP201, STM6-40/36, and STP6-120/36, using the SDM, DDM, and TDM models [16]. Convergence is obtained for the RTC cell after 500, 800 and 1000 iterations in the case of SDM, DDM and TDM, respectively and after 200 iterations for PWP201, and 2500 iterations for the other two panels.

Feng et al. proposed an improved JAYA algorithm to extract the parameters of the PV cell and panels, RTC, PWP201, STM6-40/36, and STP6-120/36 using SDM and DDM models [17]. The improvements consist of using a self-adaptive method to prevent getting stuck in a local optimum and reduce the risk of premature convergence.

The enhanced prairie dog optimizer algorithm (En-PDO) is used to extract the parameters of the RTC France photovoltaic cell and Photowatt-PWP201 panel for all three models: SDM, DDM, and TDM. Mechanisms such as random learning and logarithmic spiral search are integrated into the PDO algorithm to enhance its performance [18].

Wang et al. proposed the improved snake algorithm called multi-strategy synergistic learning snake optimizer (MSSO). The algorithm convergence is accelerated using two new strategies: the first is adaptive snake spiral foraging, and the second is dynamic update selection [19]. The MSSO was applied to the RTC France photovoltaic cell and Photowatt-PWP201 panel using only two models, DDM and TDM.

An improved version of the Coati Optimization Algorithm (COA) was achieved by Chen and Luo [20] using three strategies such as elite-guided search, horizontal crossover, and precise elimination. The new version called Multi-strategy Enhanced Coati Optimization Algorithm (MECOA) was applied to extract the parameters of the RTC France photovoltaic cell using SDM and DDM models.

Lakshmanan et al. proposed a hybrid algorithm, namely, Hybrid Golden Jackal Optimizer With Fitness Distance Balance Mechanism and Berndt-Hall-Hall-Hausman Method. This was applied to extract the parameters of the Kyocera KC200GT photovoltaic module using the TDM model. The performance of the hybrid model is proven using several metrics, among which RMSE, AE, MBE and R^2^ and comparison with several advanced hybrid algorithms [21].

The enhanced weighted-mean-of-vectors optimization algorithm (EINFO) uses an explicit objective function based on the Lambert W function to search for high-quality solutions, with Halley’s method employed for its computation [22]. This algorithm is applied to extract the parameters of the RTC France photovoltaic cell and PWP 201 photovoltaic module using SDM and DDM models.

Dev et al. applied the Marine Walrus Inspired Optimization Algorithm (WaOA) to extract the parameters of the RTC France photovoltaic cell for all three diode models, SDM, DDM and TDM. This is a hybridization between the walrus optimization metaheuristic algorithm and the Newton–Raphson iterative method [23]. For the comparison with the other six algorithms, Dev et al. used a population size of 50 and 1000 iterations. Although the algorithm converges after only 200 iterations, the execution time is quite long, more than 2100 s for 10 trials in the case of the SDM model, increasing to almost 2600 s for the TDM model.

Bakir used Fitness-distance balance-based stochastic fractal search (FDB-SFS) to estimate the parameters of the RTC France photovoltaic cell and three photovoltaic modules such as PWP 201, STP6–120/36 and STM6–40/36 [24]. The SDM and DDM models are used only for the RTC photovoltaic cell; for the modules, the SDM model is used. To prove the performance of the algorithm, RMSE is used in comparison with three algorithms: PSO, SPBO [25,26] and AGDE [27].

These algorithms and others that are outside of the considered interval, with very good results, will be used for comparison to prove the performance of the CSA and its small execution time.

## 3. Chameleon Swarm Algorithm

The Chameleon Swarm Algorithm (CSA), devised by Malik Shehadeh Braik in 2021 [28], was influenced by the characteristic foraging conduct of chameleons and their success in finding food in forests and deserts . Every metaheuristic algorithm’s efficiency depends on exploration and exploitation; hence, algorithms must be skillfully designed to balance both aspects. Comparing novel meta-heuristics against conventional methods and existing meta-heuristics, the devising of novel meta-heuristics to solve optimization issues is a systematic strategy. Exploration refers to the ability of algorithms to optimize and broadly examine various regions of the search domain; therefore, reducing the effects of local optima and escaping immobility caused by local optima is crucial. Exploitation is the capacity to find potential solutions locally across all significant domains and improve the problem-solving level.

It is vital to highlight that it is not conceivable to develop an optimization algorithm that can determine exhaustive solutions to all varieties of problems. The globular eyes of chameleons are used to scan an extensive span (360 degrees) and take advantage of every position in the search domain. Chameleons use their very long, adhesive tongues to rapidly and effectively capture prey during hunting. During the different stages of the Chameleon Swarm Algorithm, it was proposed to incorporate a customizable parameter. This parameter allows the chameleons to thoroughly investigate the problem space and attain a more optimal trade-off between exploration and exploitation. This enhancement aims to provide more consistent and dependable performance.

In addition to having exceptional eyesight that enables them to see up to 32 feet in front of them, chameleons are well-adapted to scrambling and visual hunting. Thus, this streamlines the pursuit of prey. When a potential threat is present, chameleons use their ability to alter colors to suit the situation and safeguard themselves. The mathematical models used in this work to yield CSA and carry out optimization were inspired by the foraging behavior of chameleons and their effective strategies of spotting, pursuing, and catching prey.

The main stages of a chameleon attaining victims are as follows:

1. Spotting the victims: Chameleons scout the vicinity and the natural environment for victims, but as a result, their spotting varies.
2. Stalking victims via their eyes: Each eye may be present simultaneously to swivel and focus on the victim’s location. Together, the eyes look forward, providing a binocular vision of the victim. Chameleons can see everything around them because they have a 360° viewing radius around their body and 180 degrees on each side. The chameleon’s two eyes may then focus on the same area for accurate aim if it stalks the victim. It rotates and moves towards the victim.
3. Snatching the victim: Most of the time, chameleons snare their victim with sticky tongues.

### 3.1. Mathematical Demonstration of CSA

The development of the CSA was motivated by the behavior of chameleons in identifying, pursuing, and snatching victims. In the following section, the critical processes of CSA are expressed through mathematical demonstration. Initialization and function evaluation: Every chameleon is a possible problem-solver and may be represented in a two-dimensional *x* matrix with size *q* × *k* where *q*—chameleon population, *k*—search space dimension. The vector in Equation (1) represents the location of the chameleon *s* in the solution domain at iteration *m*.

(1) $$x_{m}^{s}=\left[x_{m,1}^{s},x_{m,2}^{s},x_{m,3}^{s},\ldots ,x_{m,k}^{s}\right],$$

Let *s* = 1, 2, …, q, *m*—current iteration, *k*—problem dimension, and x^s^_m,k_—location of the sth chameleon at the kth dimension.

Equation (2) represents the generation of the initial population.

(2) $$x^{s}=B_{l}^{n}+a\times \left(B_{u}^{n}-B_{l}^{n}\right),$$

where, *x^s^*—*s*^th^ chameleon’s starting vector, $B_{u}^{n}$ and $B_{l}^{n}$—the lower and upper bounds of the exploration region in the nth dimension, and a—uniformly produced random numbers between 0 and 1.

The efficacy of the result is assessed for each fresh location a chameleon enters based on a fitness function. The current location is updated if the result effectiveness of the new location is higher than the outcome effectiveness of the existing one. It is fascinating to note that the chameleons in the Chameleon Swarm Algorithm stick to their current position if it produces better results than the new one.

Spotting the victims:

The location update process may be employed to simulate chameleon mobility while hunting.

(3) $$x_{m+1}^{s,n}=\left\{\begin{array}{l} x_{i}^{c,x}+b_{1}\left(L_{\mathrm{Best}(m)}^{s,n}-L_{\mathrm{Global}(m)}^{n}\right)a_{2}+b_{2}\left(L_{m}^{n}-x_{m}^{s,n}\right)a_{1}\;\;\;\;\;\;a_{s}\geq \rho^{\mathrm{xp}}\\ x_{i}^{c,x}+\tau \left(\left(B_{u}^{n}-B_{l}^{n}\right)a_{3}+B_{l}^{n}\right)\mathrm{sgn}\left(\mathrm{rand}-0.5\right)\;\;\;\;\;\;\;\;\;\;\;\;\;\;\;a_{s}<\rho^{\mathrm{xp}} \end{array}\right.\;,$$

where $x_{m+1}^{s,n}$—the *s*^th^ chameleon’s new location in the *n*^th^ dimension in the (*m* + 1)^th^ iteration phase; $x_{m}^{s,n}$—*s*^th^ chameleon’s current location in the *n*^th^ dimension in the *m*^th^ iteration phase; $L_{\mathrm{Best}(m)}^{s,n}$—the best location that *s*^th^ chameleon obtained yet in the *n*^th^ dimension at iteration cycle *m*; $L_{\mathrm{Global}(m)}^{n}$—any of the chameleon’s global best locations in the *n*th dimension determined yet in the *m*th iteration, b_1_ = 0.25 and b_2_ = 1.50—both are positive values that govern the ability to explore; a_1_, a_2_, a_3_—uniformly obtained random numbers between 0 and 1; a_s_—a uniformly obtained random number at index *s* between 0 and 1; ρ^xp^—the probability of the chameleon identifying prey = 0.1; sgn(rand − 0.5)—influences the course of both exploration and exploitation and may be a value of 1 or −1; τ—as observed in Equation (4), it is a parameter specified as a function of iterations that decreases with the number of iterations.

(4) τ=c1e−c2mMc3

where τ—the function of iteration decays with iteration according to Equation (4), c_1_= 1.0, c_2_ = 3.5. c_3_ = 3.0—constant value to control exploration and exploitation, *m*,*M*—current and maximum iteration numbers.

Stalking victims via their eyes:

Stalking the victim via their eyes, the strategies listed below are recommended.

- Switch the chameleon’s beginning location to the origin or the center of gravity.
- Find the rotation matrix that determines where the victim lies.
- Finally, via the rotation matrix at the center of gravity, update the chameleons’ location.
- Return the chameleons to where they originally came from.

A chameleon’s new location can be updated using the mathematical equations shown below:

(5) $$x_{m+1}^{s}=xR_{m}^{s}+{\bar{x}}_{m}^{s}$$

where $x_{m+1}^{s}$—following rotation, a chameleon’s new location, $xR_{m}^{s}$—the center of the chameleon’s current location prior to rotation, ${\bar{x}}_{m}^{s}$—the chameleon’s rotating centered coordinates in the search domain.

(6) $$xR_{m}^{s}=I\times xC_{m}^{s}$$

where $xC_{m}^{s}$—iteration *m* centering coordinates, *I*—a rotation matrix corresponding to a chameleon’s rotation.

(7) $$xC_{m}^{s}=x_{m}^{s}-{\bar{x}}_{m}^{s}$$

where $x_{m}^{s}$—the chameleons’ current location at iteration *m*.

(8) $$I=A\left(\alpha ,{\vec{V}}_{{\vec{\nu }}_{1},{\vec{\nu }}_{2}}\right)$$

where *A*—rotation matrices in the corresponding axes, *α*—Chameleon rotation angle, ${\vec{V}}_{{\vec{\nu }}_{1},{\vec{\nu }}_{2}}$—two orthonormal vectors in the *m*—dimensional search domain with *x* × 1 vector size.

*α* = *asgn*(*rand* − 0.5) × 180° (9)

where *a*—the random number between [0,1] is generated for establishing a rotation angle ranging from 0 degrees to 180 degrees. sgn(*rand* − 0.5)—the rotational direction is either 1 or 1.

The rotation matrices in tandem with the *X* and *Y* axes in three dimensions are as follows:

(10) $$I^{X}=\left[\begin{array}{c} 1\;\;\;\;\;\;0\;\;\;\;\;\;\;\;\;\;\;\;\;\;\;\;\;\;0\\ 0\;\;\;\;\;\cos\delta_{X}\;\;\;\;\;\;-\sin\delta_{X}\\ 0\;\;\;\;\;\sin\delta_{X}\;\;\;\;\;\;\;\;\;\;\cos\delta_{X} \end{array}\right]$$

(11) $$I^{Y}=\left[\begin{array}{c} \cos\delta_{Y}\;\;\;\;\;\;\;\;\;\;\;0\;\;\;\;\;\;\;\;\;\;\;\;\;\;\sin\delta_{Y}\\ 0\;\;\;\;\;\;\;\;\;\;\;\;\;\;\;\;\;\;\;1\;\;\;\;\;\;\;\;\;\;\;\;\;\;0\\ -\sin\delta_{Y}\;\;\;\;\;\;\;0\;\;\;\;\;\;\;\;\;\;\;\;\;\;\cos\delta_{Y} \end{array}\right]$$

where *δ_X_*—the rotational angle pertaining to the *X*-axis, *δ_Y_*—the rotational angle pertaining to the *Y*-axis.

Snatching the victim: The best chameleon is the one that approaches its victim as closely as possible, since this is optimal. The chameleon uses a tongue to chase the victim. Its location is somewhat altered as a result, since it may extend its tongue more than twice its length.

The tongue velocity of a chameleon is calculated using Equation (12)

(12) $$T_{m+1}^{s,n}=\beta T_{m}^{s,n}+d_{1}\left(L_{\mathrm{Global}(m)}^{s,n}-x_{m}^{s,n}\right)a_{4}+d_{2}\left(L_{\mathrm{Best}(m)}^{s,n}-x_{m}^{s,n}\right)a_{5}$$

where $T_{m+1}^{s,n}$—the *s*^th^ chameleon’s new velocity in the *n*^th^ dimension at iteration *m* + 1, $T_{m}^{s,n}$—the *s*^th^ chameleon’s current velocity in the *n*^th^ dimension at iteration *m*, $x_{m}^{s,n}$—the *s*^th^ chameleon’s current location, $L_{\mathrm{Best}(m)}^{s,n}$—the *s*^th^ chameleon’s best known location, $L_{\mathrm{Global}(m)}^{s,n}$—best global location attained yet by the chameleons, *d*_1_, *d*_2_—constant values equal to 1.75 and 1.75, respectively, control the effect of $L_{\mathrm{Best}(m)}^{s,n}$ and $L_{\mathrm{Global}(m)}^{s,n}$ on the chameleon’s tongue dropping, *a*_4_, *a*_5_—two random numbers dispersed range 0 and 1, *β*—the inertia weight that decays linearly with consecutive iterations according to Equation (13). The behavior of the CSA is improved.

(13) β=1−mMmMe1

where *e*_1_—positive value equal to one, used to regulate exploitation ability, *m*,*M*—current and maximum iteration numbers.

*z* = 2590 × (1 − *e*^−*log*(*m*)^) (14)

The third equation of motion may be used to determine the chameleon’s location,

(15) xm+1s,n=xms,n+Tms,n2−Tm−1s,n22z

where $T_{m-1}^{s,n}$—the *s*^th^ chameleon’s previous velocity in the *n*^th^ dimension, *z*—the rate at which the chameleon’s tongue projection accelerates, which is described in Equation (14).

**Figure 2:**
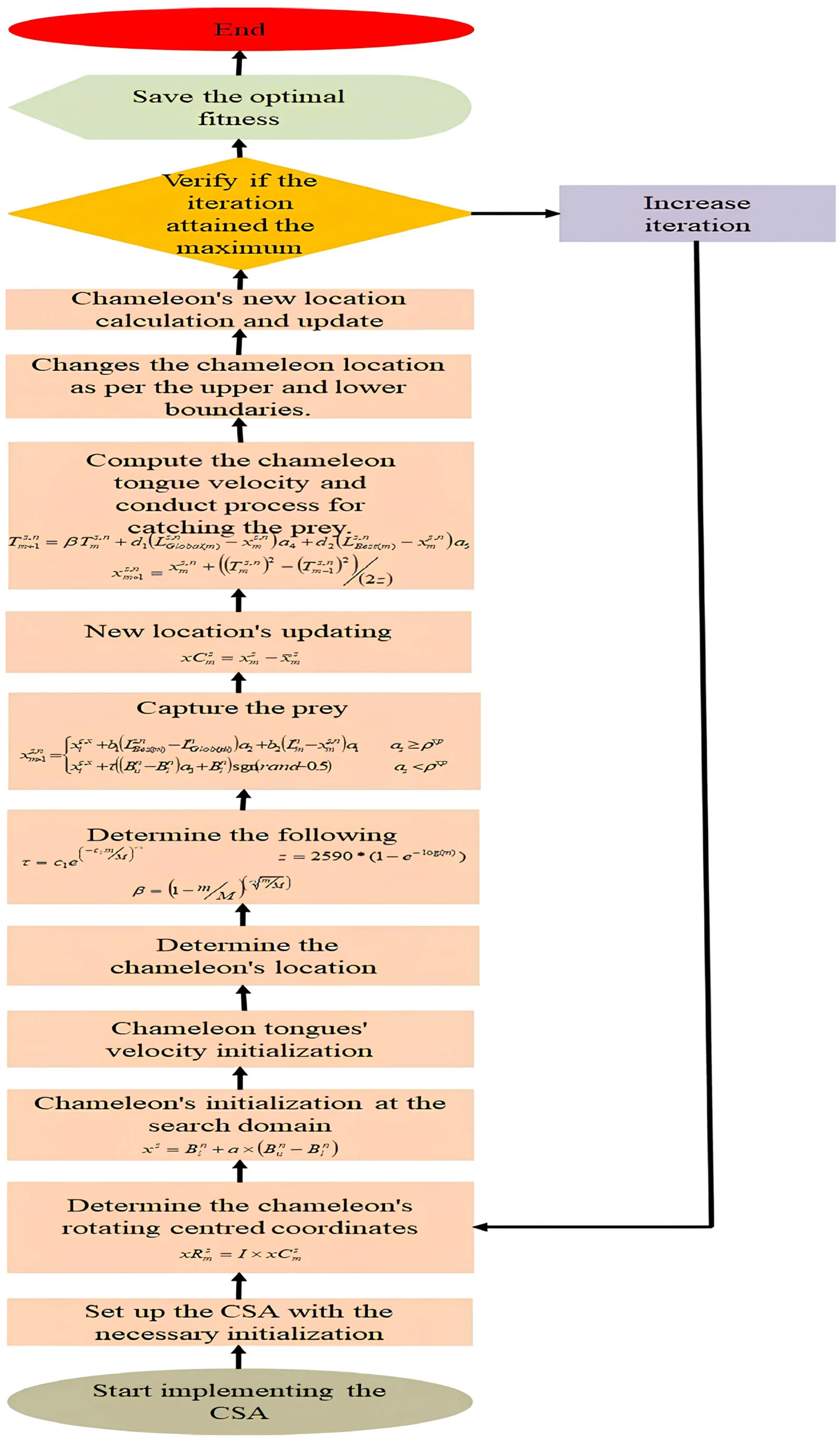
Process and stages of CSA.

Equations (3), (5) and (15) were introduced to represent the stalking behaviors of the chameleon. The process and stages can be seen via an illustration in Figure 2.

### 3.2. Chameleon Swarm Algorithm Process

The following are the stages in the Chameleon Swarm Algorithm (CSA) process:

Stage 1: Initialize all the CSA’s necessary variable parameters.
Stage 2: Apply Equation (6) to determine the chameleon’s rotating centered coordinates.
Stage 3: Apply Equation (2) to initialize the chameleon in the search domain.
Stage 4: Set up the chameleon tongues’ starting velocity
Stage 5: Determine the chameleon’s location.
Stage 6: Calculate Equations (4), (13) and (14)
Stage 7: Using the Equation, spotting the victims (3)
Stage 8: Use Equation (5) to update the new location.
Stage 9: Carry out the process of snatching the victim.
Stage 10: Use Equations (12) and (15) to calculate the chameleon tongue’s velocity and location.
Stage 11: The chameleon location is modified based on the upper and lower bounds.
Stage 12: Determine and update the chameleon’s new location.

## 4. Models for Photovoltaic Devices

All three models that are used to characterize the photovoltaic cells and modules are well-known and derive from the corresponding equivalent circuits [18]. In the following, only the mathematical equations for the three models for photovoltaic cells are given: SDM Equation (16), DDM Equation (17), and TDM Equation (18).

(16) $$I=I_{\mathrm{ph}}-I_{o}\left(e^{\frac{V+IR_{s}}{{\mathrm{nV}}_{T}}}-1\right)-\frac{V+IR_{s}}{R_{\mathrm{sh}}},V_{T}=\frac{\mathrm{kT}}{q}$$

where *I_ph_* is the photogenerated current, *I_o_* is the reverse saturation current, *R_s_* is the series resistance, *R_sh_* is the shunt resistance, *n* is the ideality factor of the diode, *V_T_* is the thermal voltage, *T* is the cell or module temperature, *k =* 1.3806503 × 10^−23^ is the Boltzmann constant, and *q =* 1.60217646 × 10^−19^ is the elementary electrical charge [29].

(17) $$I=I_{\mathrm{ph}}-I_{o1}\left(e^{\frac{V+IR_{s}}{{n_{1}V}_{T}}}-1\right)-I_{o2}\left(e^{\frac{V+IR_{s}}{{n_{2}V}_{T}}}-1\right)-\frac{V+IR_{s}}{R_{\mathrm{sh}}}$$

(18) $$I=I_{\mathrm{ph}}-I_{o1}\left(e^{\frac{V+IR_{s}}{{n_{1}V}_{T}}}-1\right)-I_{o2}\left(e^{\frac{V+IR_{s}}{{n_{2}V}_{T}}}-1\right)-I_{o3}\left(e^{\frac{V+IR_{s}}{{n_{3}V}_{T}}}-1\right)-\frac{V+IR_{s}}{R_{\mathrm{sh}}}$$

where index 1 is for the diffusion mechanism, 2 is for the generation and recombination mechanism, and 3 is for the thermionic process with recombination in the defect area.

In the case of the photovoltaic module with N_s_ cells connected in series, Equation (19) shows the mathematical model for the SDM model. For the other two, the DDM and TDM models, the mechanism is the same as that of the cell.

(19) $$I=I_{\mathrm{ph}}-I_{o}\left(e^{\frac{V+N_{s}IR_{s}}{{nN_{s}V}_{T}}}-1\right)-\frac{V+N_{s}IR_{s}}{N_{s}R_{\mathrm{sh}}}$$

The objective function is considered RMSE [30] because the purpose is to minimize the error between the calculated and measured data based on the I-V characteristic, Equation (20) [31].

(20) $$\mathrm{RMSE}(x)=\sqrt{\frac{{\sum_{i=1}^{N}f_{k}\left(V_{i},I_{i},x\right)}^{2}}{N}}$$

where N is the number of (V,I) pairs, k is the considered diode model, and x is the parameter vector.

The error functions for the three models are given by the following equations: SDM (21), DDM (22) and TDM (23) [32]

(21) $$\left\{\begin{array}{c} f_{\mathrm{SDM}}\left(V,I,x\right)=I_{\mathrm{ph}}-I_{o}\left(e^{\frac{V+IR_{s}}{{\mathrm{nV}}_{T}}}-1\right)-\frac{V+IR_{s}}{R_{\mathrm{sh}}}-I\\ x=\left(I_{\mathrm{ph}},I_{o},R_{s},R_{\mathrm{sh}},n\right) \end{array}\right.$$

(22) $$\left\{\begin{array}{c} f_{\mathrm{DDM}}\left(V,I,x\right)=I_{\mathrm{ph}}-I_{o1}\left(e^{\frac{V+IR_{s}}{{n_{1}V}_{T}}}-1\right)-I_{o2}\left(e^{\frac{V+IR_{s}}{{n_{2}V}_{T}}}-1\right)-\frac{V+IR_{s}}{R_{\mathrm{sh}}}-I\\ x=\left(I_{\mathrm{ph}},I_{o1},{I_{o2},R}_{s},R_{\mathrm{sh}},n_{1},n_{2}\right) \end{array}\right.$$

(23) $$\left\{\begin{array}{c} f_{\mathrm{TDM}}\left(V,I,x\right)=I_{\mathrm{ph}}-I_{o1}\left(e^{\frac{V+IR_{s}}{{n_{1}V}_{T}}}-1\right)-I_{o2}\left(e^{\frac{V+IR_{s}}{{n_{2}V}_{T}}}-1\right)-I_{o3}\left(e^{\frac{V+IR_{s}}{{n_{3}V}_{T}}}-1\right)-\frac{V+IR_{s}}{R_{\mathrm{sh}}}-I\\ x=\left(I_{\mathrm{ph}},I_{o1},{I_{o2},I_{o3},R}_{s},R_{\mathrm{sh}},n_{1},n_{2},n_{3}\right) \end{array}\right.$$

## 5. Results and Discussion

The Chameleon Swarm Algorithm was used to extract the parameters for the multijunction solar cell [33]. Now, it is used to extract parameters of two photovoltaic cells and one module.

The experiments were implemented and executed in MATLAB R2025b on a 64-bit Microsoft Windows 11 Home operating system. The computations were performed on an Intel Core 9, 10 Core (s), 3.6 GHz, 20 MB; GPU: NVIDIA GeForce RTX 3080Ti 12 GB; RAM: 32. The system uses UEFI firmware and x64-based architecture.

The performance of the CSA in parameter extraction was compared with that of other algorithms reported in the specialized literature. The current and power voltage characteristics were measured at 1000 W/m^2^ and different temperatures for the devices under test.

There are established lower and upper bound (LB and UB) values for the parameters of the photovoltaic cells and modules to have a fair comparison; see Table 1.

Six statistical metrics are employed to evaluate the performance of the CSA, namely the root mean square error (RMSE) which shows the difference between measured and calculated values for the current, Equation (24), the mean absolute error (MAE) that give a measure of the average errors, Equation (25), the mean bias error (MBE) which gives a measure of the average tendency of a model to overestimate or underestimate real values, Equation (26), the mean relative error (MRE) measures the average relative errors between calculated and measured current values, Equation (27), the mean squared error (MSE) shows the average squared difference between calculated values for the current of the PV and measured values; a lower value indicates higher accuracy in estimating, Equation (28), and the coefficient of determination that shows if the matching between the calculated current and the measured current is high, the best value is 1, Equation (29).

(24) $$\mathrm{RMSE}=\sqrt{\frac{{\sum_{i=1}^{N}\left(I_{\mathrm{mi}}-I_{\mathrm{ci}}\right)}^{2}}{N}}$$

(25) $$\mathrm{MAE}=\frac{\sum_{i=1}^{N}\left|I_{\mathrm{mi}}-I_{\mathrm{ci}}\right|}{N}$$

(26) $$\mathrm{MBE}=\frac{\sum_{i=1}^{N}\left(I_{\mathrm{mi}}-I_{\mathrm{ci}}\right)}{N}$$

(27) $$\mathrm{MRE}=\frac{\sum_{i=1}^{N}\left|\frac{I_{\mathrm{mi}}-I_{\mathrm{ci}}}{I_{\mathrm{mi}}}\right|}{N}$$

(28) $$\mathrm{MSE}=\frac{\sum_{i=1}^{N}{\left(I_{\mathrm{mi}}-I_{\mathrm{ci}}\right)}^{2}}{N}$$

(29) $$R^{2}=1-\frac{\sum_{i=1}^{N}{\left(I_{\mathrm{mi}}-I_{\mathrm{ci}}\right)}^{2}}{\sum_{i=1}^{N}{\left(I_{\mathrm{mi}}-\bar{I_{\mathrm{mi}}}\right)}^{2}}$$

### 5.1. RTC France Photovoltaic Cell

The I-V characteristic was measured at 33 °C, and 26 (V,I) pairs were considered. This dataset is the most widely used in the specialized literature to demonstrate the performance of algorithms and methods for extracting parameters of photovoltaic cells and modules [33,34].

To evaluate the stochastic behavior of CSA, 20 independent runs are added for every model and iteration setting. Each run uses a distinct random seed. For each run, the parameter vector yielding the lowest RMSE is retained.

The five, seven and nine parameters of the RTC photovoltaic cell are presented in Table 2. These parameters are obtained using the CSA with 250 search agents and various iterations from 1000 to 2000 in increments of 250.

Extracting the parameters of a photovoltaic device is a multimodal problem. The objective function contains local minima where the algorithm can get trapped. If the global minimum is not reached, different parameter solutions may exist that yield very similar RMSE values. For example, for the TDM model with 1500 iterations, the RMSE value is 9.7959621 × 10^−4^—a value very close to the RMSE for 2000 iterations, which is 9.7805717 × 10^−4^. The values of the reverse saturation currents and the ideality factor of diodes for the three mechanisms show significant differences Table 2.

The distributions of the parameters of the RTC photovoltaic cell across the 20 runs are summarized using mean, median, standard deviation, minimum, maximum, interquartile range (IQR), see Equation (30) and coefficient of variation, Equation (31). Table S1 presents these statistical metrics for the extracted parameters of the RTC cell utilizing the SDM, DDM, and TDM models. Only the results when 2000 iterations were applied, yielding the lowest RMSE value, are shown.

(30) $$\mathrm{IQR}=Q_{3}-Q_{1},\;\left\{\begin{array}{c} Q_{1}=25\mathrm{th}\;\mathrm{percentile}\;\mathrm{of}\;\mathrm{the}\;\mathrm{metric}\;\mathrm{across}\;\mathrm{runs}\\ Q_{3}=75\mathrm{th}\;\mathrm{percentile}\;\mathrm{of}\;\mathrm{the}\;\mathrm{metric}\;\mathrm{across}\;\mathrm{runs} \end{array}\right.$$

(31) $$\mathrm{CV}=\frac{\sigma }{\bar{x}}100,\;\sigma \;\mathrm{is}\;\mathrm{standard}\;\mathrm{deviation},\;\bar{x\;}\;\mathrm{is}\;\mathrm{the}\;\mathrm{average}\;\mathrm{of}\;x$$

IQR for all parameters is the lowest for the SDM model, which means the middle values are close together and more consistent. The highest are for the DDM model, so there is high variability.

The analysis of the results presented in Table S1 for the SDM model reveals very low values for standard deviations, interquartile ranges, and coefficients of variation obtained from independent optimization runs. This demonstrates a high degree of stability and reproducibility in parameter estimation. The results indicate that the proposed identification method consistently converges with the same set of parameters. In the case of the DDM model, *I_ph_*, *R_s_*, *n*_1_, and *R_sh_* exhibit high stability. In contrast, *I_o_*_1_ and *n*_2_ show considerably higher variability, while *I_o_*_2_ exhibits the highest variability. In the case of the TDM model, *I_ph_*, *R_s_*, and *R_sh_* exhibit high stability. In contrast, the diode-related parameters show substantially larger variations.

**Figure 3:**
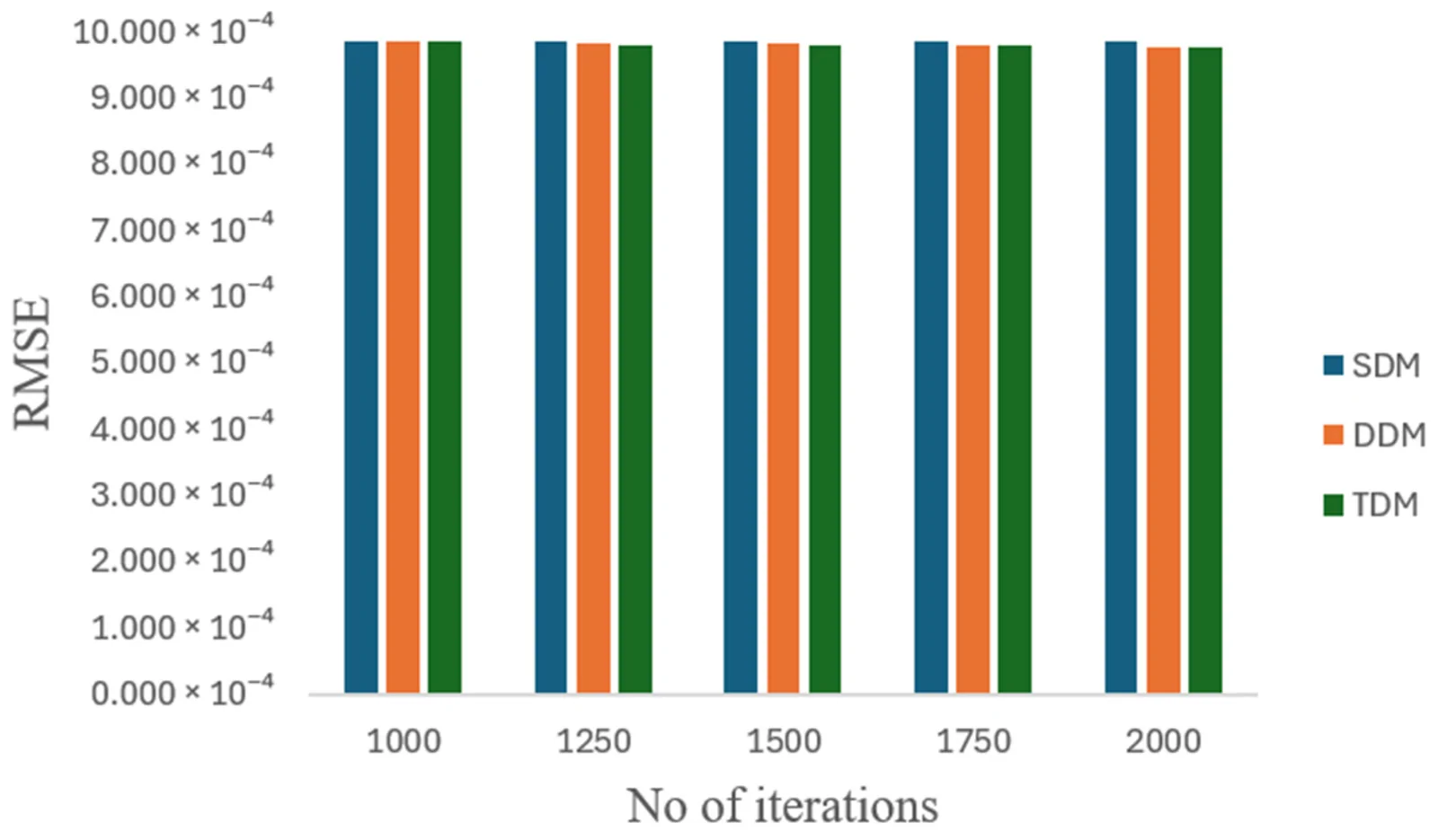
RMSE vs. iterations for the RTC photovoltaic cell.

The most commonly used statistical metric in analyzing parameter extraction is the RMSE. Figure 3 shows the variation in RMSE for different numbers of iterations and the same number of populations, 250. A pattern can be observed, namely the increase in the number of iterations proportionally with the accuracy of determining the parameters. For the SDM model, the lowest value of RMSE is 9.8602188 × 10^−4^, obtained for all considered iterations, except for 1000. In the case of DDM, the lowest value is obtained for 2000, namely, 9.767 × 10^−4^. Also, the lowest RMSE for the TDM model is obtained for 2000 iterations, and it is 9.78057 × 10^−4^. The conclusion of the study on variation in the number of iterations is that the algorithm provides very good results for a number greater than or equal to 1500. Below this threshold, performance drops across all three models: SDM, DDM, and TDM.

**Figure 4:**
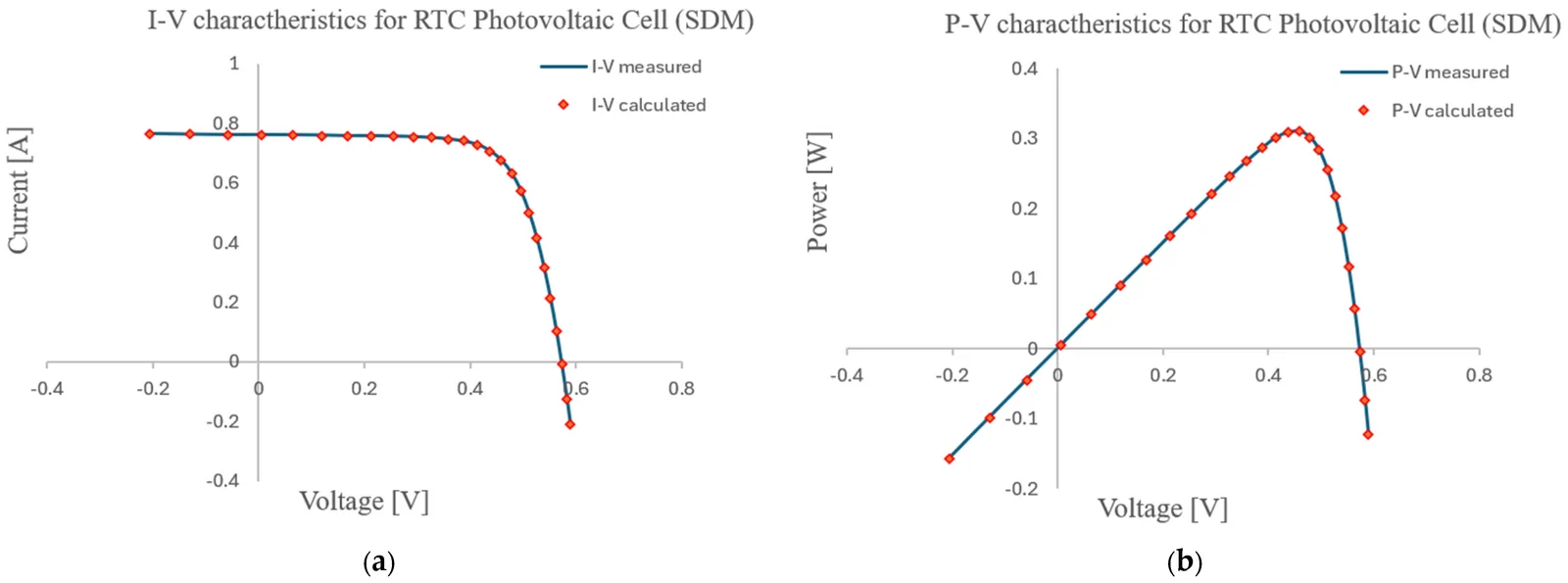
RTC photovoltaic cell characteristics measured vs. calculated—SDM: (**a**) I-V characteristic; (**b**) P-V characteristic.

**Figure 5:**
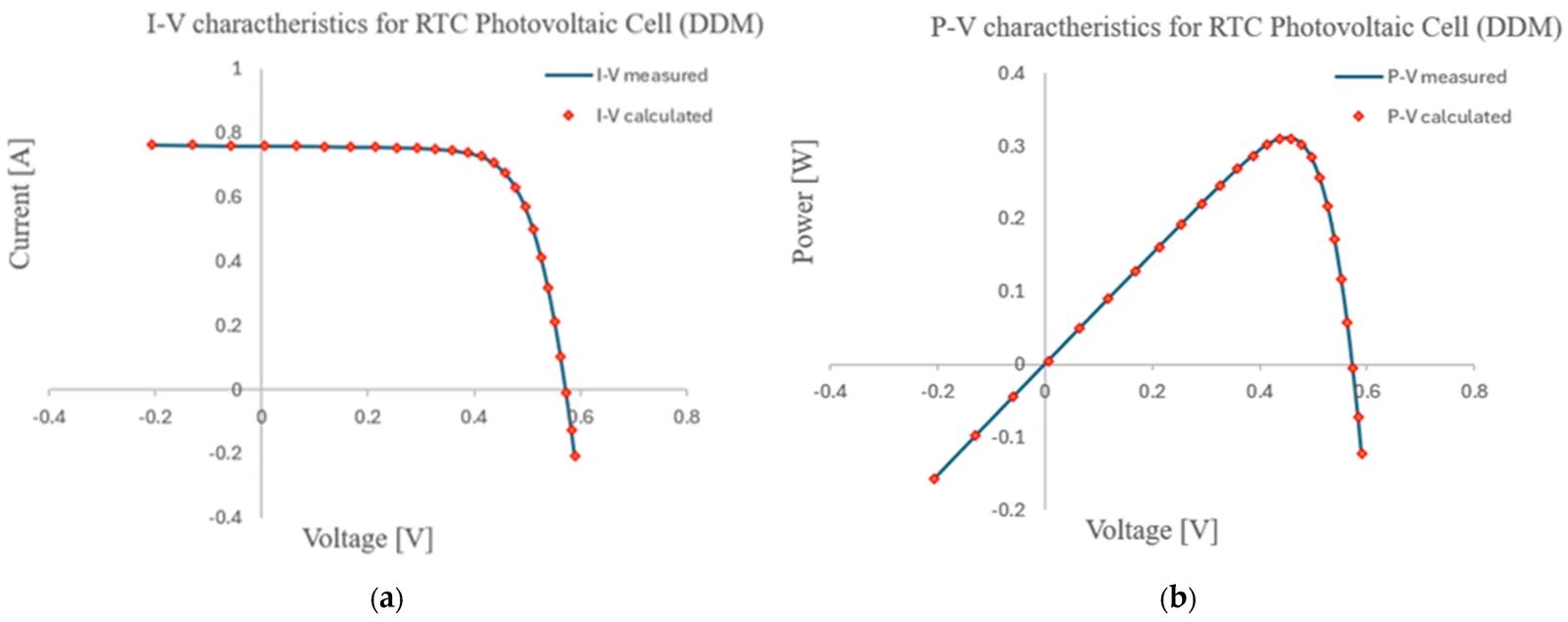
RTC photovoltaic cell characteristics measured vs. calculated—DDM: (**a**) I-V characteristic; (**b**) P-V characteristic.

**Figure 6:**
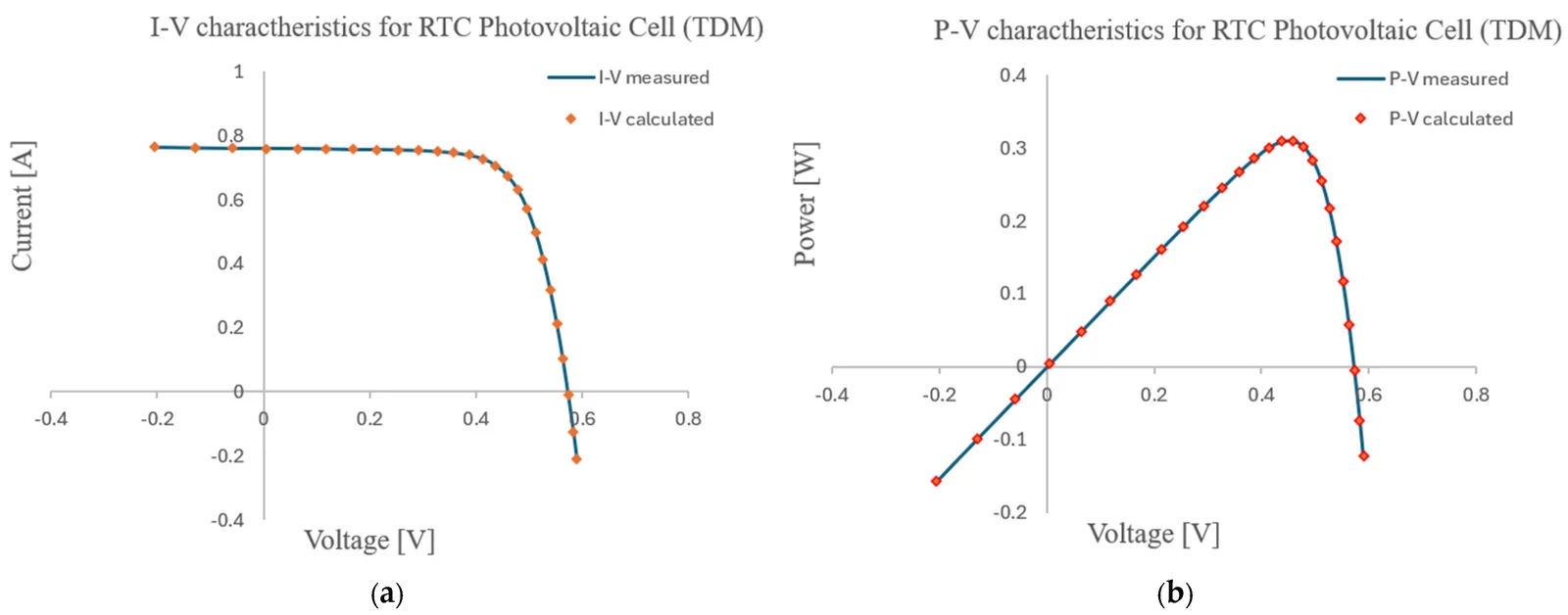
RTC photovoltaic cell characteristics measured vs. calculated—TDM: (**a**) I-V characteristic; (**b**) P-V characteristic.

The very good matching between the measured and calculated values using the CSA for the I-V and P-V characteristics is found in Figure 4 in the case of the SDM model, Figure 5 for the DDM model, and Figure 6 for the TDM model. This proves an excellent estimation of the RTC parameters, confirmed also by Table 3.

**Figure 7:**
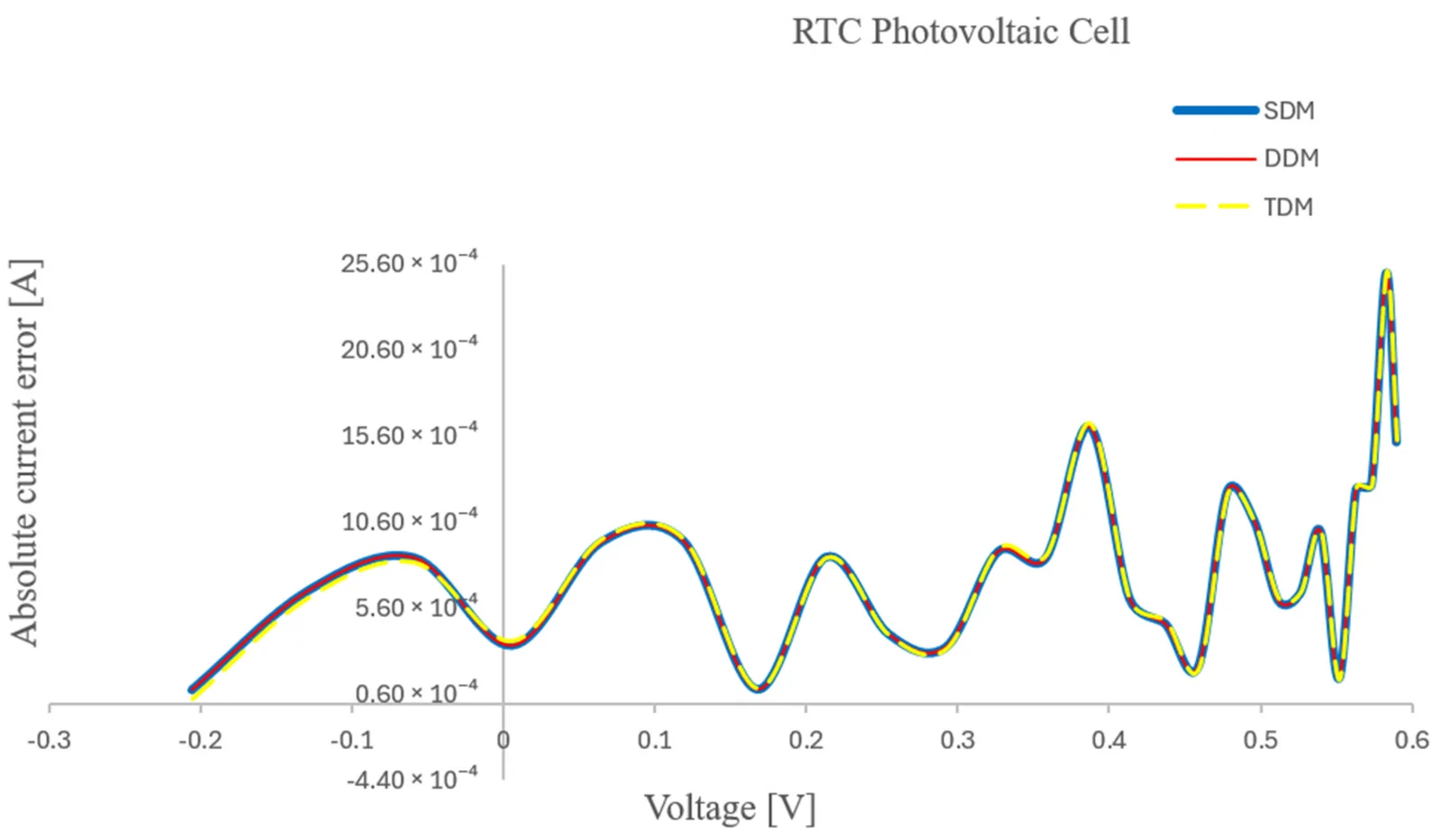
Absolute current error comparison for the three diode models for the RTC France photovoltaic cell.

The absolute current errors for the three diode models are presented in Figure 7. The differences between the three models are relatively small. For better visualization, these values are presented in the supplementary files in Table S2, which contains the measured voltage and current values, as well as the calculated current for the SDM, DDM, and TDM models.

**Figure 8:**
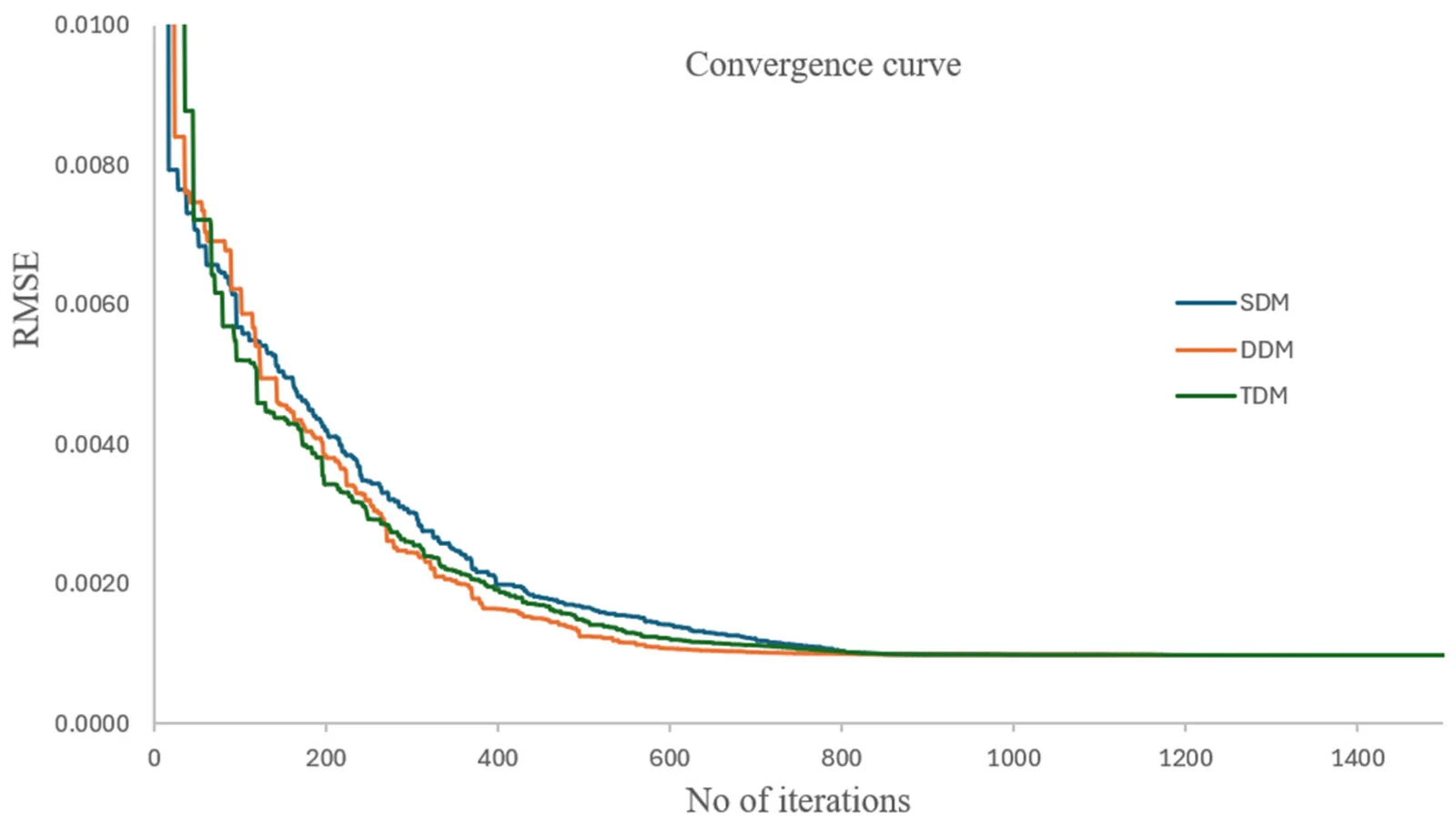
Convergence curves of CSA for the RTC photovoltaic cell.

Figure 8 shows the convergence curve for the CSA for the three models used in the parameter extraction for the RTC photovoltaic cell. The DDM model has the best performance in terms of convergence speed, followed by the TDM model and then the SDM model.

A comparison between the CSA and several algorithms, recent and among the best, from the specialized literature is presented in Table 3. To make this comparison, six statistical metrics are used, and their values are calculated using the parameter values taken from the papers. Analyzing the results of the statistical metrics shows that the CSA has very good performance in the extraction of the parameters of the RTC photovoltaic cell. In the case of the SDM model, the RMSE has one of the lowest values in comparison with the other fifteen algorithms considered. Also, this occurs for MSE and R2. The best values for RMSE, MSE, and R2 are obtained for the DDM and TDM models. CSA proves its performance for all three models, especially for the DDM and TDM models.

Shaban et al. applied the Runge–Kutta Optimization algorithm (RUN) to extract the parameters of the RTC photovoltaic cell. To evaluate the performance of RUN, it was compared against CSA and five other algorithms. The results for CSA (labeled as CSAo in Table 3 were poor. Furthermore, for the TDM model, its parameter values were practically unusable, resolving to either 1 or 0 [35].

**Table 3:** Values of the statistical metrics for the RTC photovoltaic cell.

Models	Algoritms	RMSE [10^−4^]	MAE [10^−4^]	MBE	MRE	MSE	R^2^
SDM	**CSA**	**9.86021**	**8.27956**	**−1.29816** × **10^−5^**	**7.39237** × **10^−3^**	**9.72239** × **10^−7^**	**0.9999893**
	I-CPA	9.98621	8.14517	4.10747 × 10^−5^	6.79639 × 10^−3^	9.97855 × 10^−7^	0.9999893
	ISOA	9.86029	8.27873	1.54925 × 10^−8^	7.40163 × 10^−3^	9.72254 × 10^−7^	0.9999893
	HSDA	9.86022	8.27989	1.42971 × 10^−5^	7.39898 × 10^−3^	9.73421 × 10^−7^	0.9999893
	IAPO	9.86022	8.27995	−4.77295 × 10^−7^	7.39399 × 10^−3^	9.72239 × 10^−7^	0.9999893
	CSAO	9.86287	8.31378	−1.29276 × 10^−5^	7.56726 × 10^−3^	9.72763 × 10^−7^	0.9999891
	SJAYA	9.86104	8.26045	6.7051 × 10^−6^	7.29420 × 10^−3^	9.72402 × 10^−7^	0.9999891
	FDB-SFS	9.86023	8.27973	1.49123 × 10^−8^	7.40012 × 10^−3^	9.72134 × 10^−7^	0.9999893
	En-PDO	9.95213	8.42281	−6.19851 × 10^−5^	7.96193 × 10^−3^	9.90449 × 10^−7^	0.9999891
	MECOA	9.86338	8.31736	−1.43878 × 10^−5^	7.58282 × 10^−3^	9.72863 × 10^−7^	0.9999892
	EINFO	10.2301	8.11038	1.15512 × 10^−4^	5.26822 × 10^−3^	1.04655 × 10^−6^	0.9999884
	WaOA	9.89085	8.26648	−2.33336 × 10^−4^	7.15484 × 10^−3^	9.78289 × 10^−7^	0.9999892
	SFOA [34]	9.87964	8.37200	−3.72302 × 10^−5^	7.86342 × 10^−3^	9.76074 × 10^−7^	0.9999892
	CSAo [36]	25.7785	20.5291	5.31537 × 10^−5^	1.7543 × 10^−2^	6.64531 × 10^−6^	0.9999269
	ECO-Secant [37]	9.95405	8.41112	−6.63271 × 10^−5^	7.93998 × 10^−3^	9.90833 × 10^−7^	0.9999891
	HSO [38]	10.5512	8.62321	−2.23452 × 10^−5^	7.82341 × 10^−3^	1.13446 × 10^−6^	0.9999872
	ICSE [39]	9.86022	8.27959	−8.61057 × 10^−5^	7.39463 × 10^−3^	9.97855 × 10^−7^	0.9999893
DDM	**CSA**	**9.76700**	**8.11533**	**1.94415** × **10^−7^**	**7.02919** × **10^−3^**	**9.53943** × **10^−7^**	**0.9999895**
	I-CPA	10.6944	8.64148	1.14282 × 10^−6^	7.71015 × 10^−3^	1.14372 × 10^−6^	0.9999874
	ISOA	9.82157	8.19554	3.6554 × 10^−8^	8.00673 × 10^−3^	9.64633 × 10^−7^	0.9999894
	IAPO	9.82484	8.18295	−8.48123 × 10^−8^	7.78418 × 10^−3^	9.65276 × 10^−7^	0.9999894
	HSDA	9.82174	8.16746	−3.30237 × 10^−6^	7.29956 × 10^−3^	9.61882 × 10^−7^	0.9999893
	MSSO	10.1469	8.63231	−5.13547 × 10^−6^	7.88035 × 10^−3^	1.02961 × 10^−6^	0.9999886
	MECOA	9.82523	8.19642	−5.25528 × 10^−6^	7.87078 × 10^−3^	9.65353 × 10^−7^	0.9999893
	FDB-SFS	9.84123	8.11213	−4.96123 × 10^−6^	7.65713 × 10^−3^	9.68351 × 10^−7^	0.9999893
	SFOA [34]	9.87742	8.09097	4.74173 × 10^−5^	7.03585 × 10^−3^	9.75635 × 10^−7^	0.9999892
	ECO-Secant [37]	9.96555	8.35791	−7.40706 × 10^−5^	8.88444 × 10^−3^	9.93123 × 10^−7^	0.9999890
	HSO [38]	10.1012	8.32425	−4.32413 × 10^−5^	7.12132 × 10^−3^	1.03452 × 10^−6^	0.9999883
	ICSE [39]	9.82485	8.18278	−9.32695 × 10^−6^	7.34233 × 10^−3^	9.75481 × 10^−7^	0.9999893
	CSAo [36]	119.2120	82.8057	−6.67039 × 10^−3^	2.09888 × 10^−2^	1.42115 × 10^−4^	0.9984368
TDM	**CSA**	**9.78057**	**8.15704**	**2.76141** × **10^−6^**	**7.17376** × **10^−3^**	**9.56595** × **10^−7^**	**0.9999895**
	CSAO	9.82527	8.19661	−5.00408 × 10^−6^	7.87600 × 10^−3^	9.65361 × 10^−7^	0.9999893
	En-PDO	10.0154	8.18524	−5.33835 × 10^−5^	7.74796 × 10^−3^	1.00308 × 10^−6^	0.9999889
	WaOA	10.1287	8.17819	−2.33701 × 10^−6^	6.45175 × 10^−3^	1.02592 × 10^−6^	0.9999887
	SFOA [31]	9.82465	8.20275	2.01617 × 10^−5^	7.90082 × 10^−3^	9.65239 × 10^−7^	0.9999893
	ECO-Secant [37]	10.5495	8.42181	6.86731 × 10^−5^	7.75844 × 10^−3^	1.11293 × 10^−6^	0.9999877

The values for the following metrics of the RMSE, such as mean, standard deviation, minimum, maximum, and interquartile range, are shown in Table 4. For the CSA, they are calculated after the algorithm runs 20 times. The results for the other algorithms are considered from specialized literature; the comparison has some limitations due to differences in population sizes, iteration counts, stopping criteria, and numbers of runs for the references. In the case of the SDM model, the mean, standard deviation, minimum, and maximum of RMSE are better than those of the other algorithms considered. For IAPO and SJAYA, the mean, minimum, and maximum are the same as CSA, but the standard deviation is slightly lower, which means the CSA is more stable. In the case of the DDM model, CSA has the lowest minimum value, but the mean and standard deviation are slightly lower for the CSOA and SJAYA than for CSA, which means that CSOA and SJAYA are more robust. In the case of the TDM, the CSA is more robust than CSOA and *μ*AFCSO because all metrics are better.

### 5.2. aSi:H Photovoltaic Cell

The I-V characteristic was measured at 26 °C, and 46 (V,I) pairs were considered. This dataset was measured at the Idonial R&TD center—Surfaces and coatings. The I-V characteristic was measured with a 4200A Keitlhey analyzer, West Yorkshire, United Kingdom, and the aSi was illuminated using a LOT Oriel class A solar simulator, Darmstadt, Germany.

The five, seven and nine parameters of the aSi:H photovoltaic cell are presented in Table 5. These parameters are obtained using the CSA with 250 search agents and various iterations from 1000 to 2000 in increments of 250.

Because the PV parameter extraction is a multimodal problem, the objective function can have local minima. If the global minimum is not reached, different parameter solutions may exist that yield similar RMSE values. For example, for the TDM model with 1500 iterations, the RMSE value is 4.1047226 × 10^−5^—a value very close to the RMSE for 2000 iterations, which is 4.094393 × 10^−5^. The values of the reverse saturation currents and the ideality factor of diodes for the three mechanisms are very different, Table 5.

The distributions of the parameters of the aSi:H photovoltaic cell across the 20 runs were summarized using the mean, median, standard deviation, minimum, maximum, interquartile range and coefficient of variation is presented in Table S3. Only the results when 1750 iterations were applied, yielding the lowest RMSE value, are shown. *I_ph_, I_o_*, *n*_1_ and *R_sh_* have extremely low standard deviations, narrow interquartile ranges, and small coefficients of variation in the case of the SDM model. This indicates negligible variation among repeated parameter estimations. *R_s_* exhibits a high coefficient of variation, as its value is very close to 0 and the coefficient of variation is highly sensitive to very small absolute variations. In the case of DDM model, *I_ph_* and *R_sh_* are identified with high precision, whereas *n*_1_ and *n*_2_ are relatively well-identified, and *R_s_, I_o_*_1_ *and I_o_*_2_ show lower identifiability. The results indicate in the case of TDM model that *I_ph_* and *R_sh_* are robust, whereas *R_s_* and the diode-related parameters are more sensitive to the optimization process and may exhibit parameter compensation or correlation.

**Figure 9:**
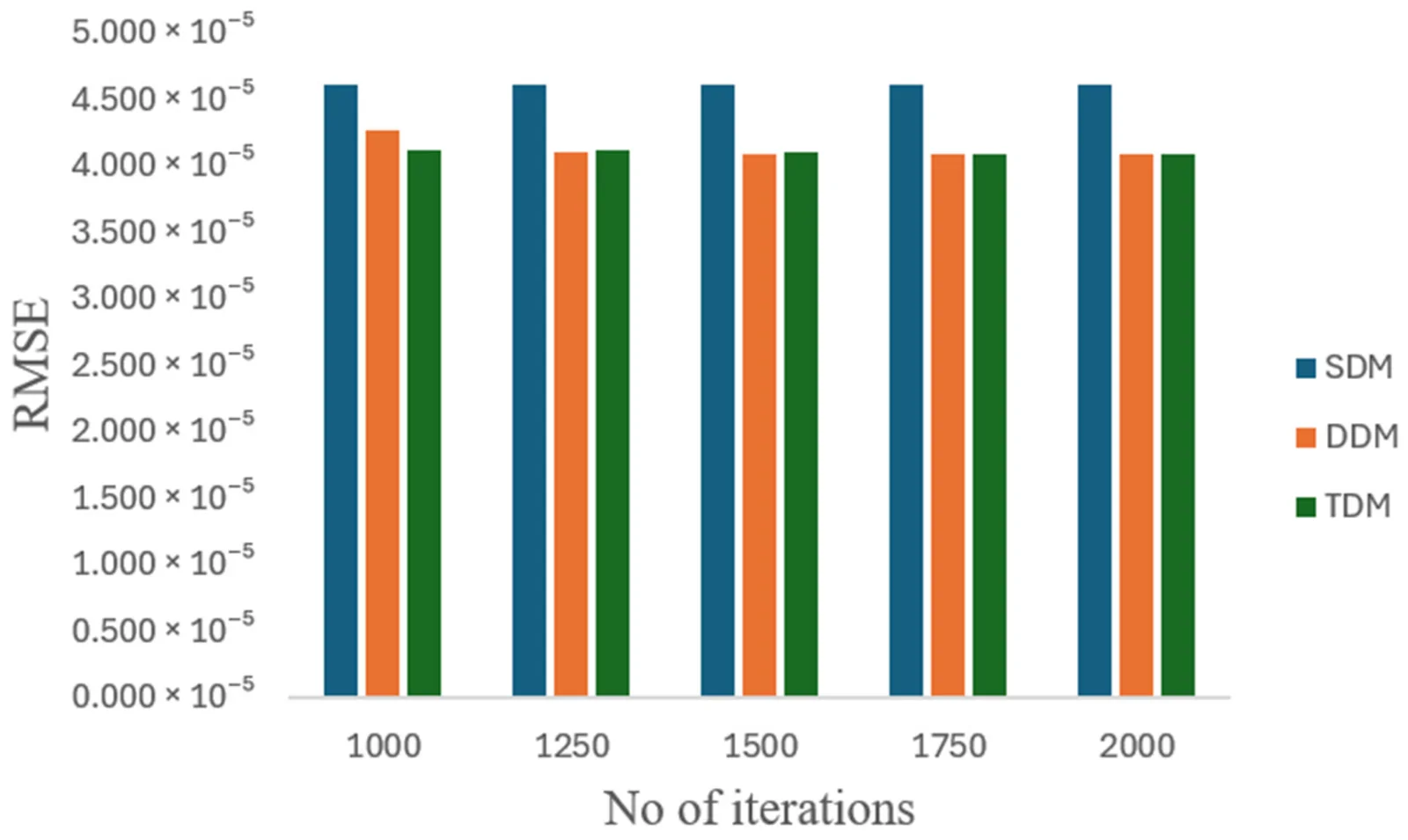
RMSE vs. iterations for the aSi:H photovoltaic cell.

The most used statistical test is the RMSE in analyzing parameter extraction. Figure 9 shows the variation in the RMSE for different numbers of iterations and the same number of populations—250. There is a pattern, as can be observed. The RMSE decreases when transitioning from SDM to DDM and then to TDM. For SDM model the value of RMSE is 4.612321 × 10^−5^ from 1250 iterations to 2000, respectively. In the case of DDM and TDM, as the number of iterations increases, the RMSE decreases to 4.0949367 × 10^−5^. and 4.094393 × 10^−5^. respectively—values recorded at 1750 iterations, representing the best result—before rising slightly at 2000 iterations. The conclusion of the study of variation in the number of iterations is that the algorithm provides very good results for 1750 iterations.

**Figure 10:**
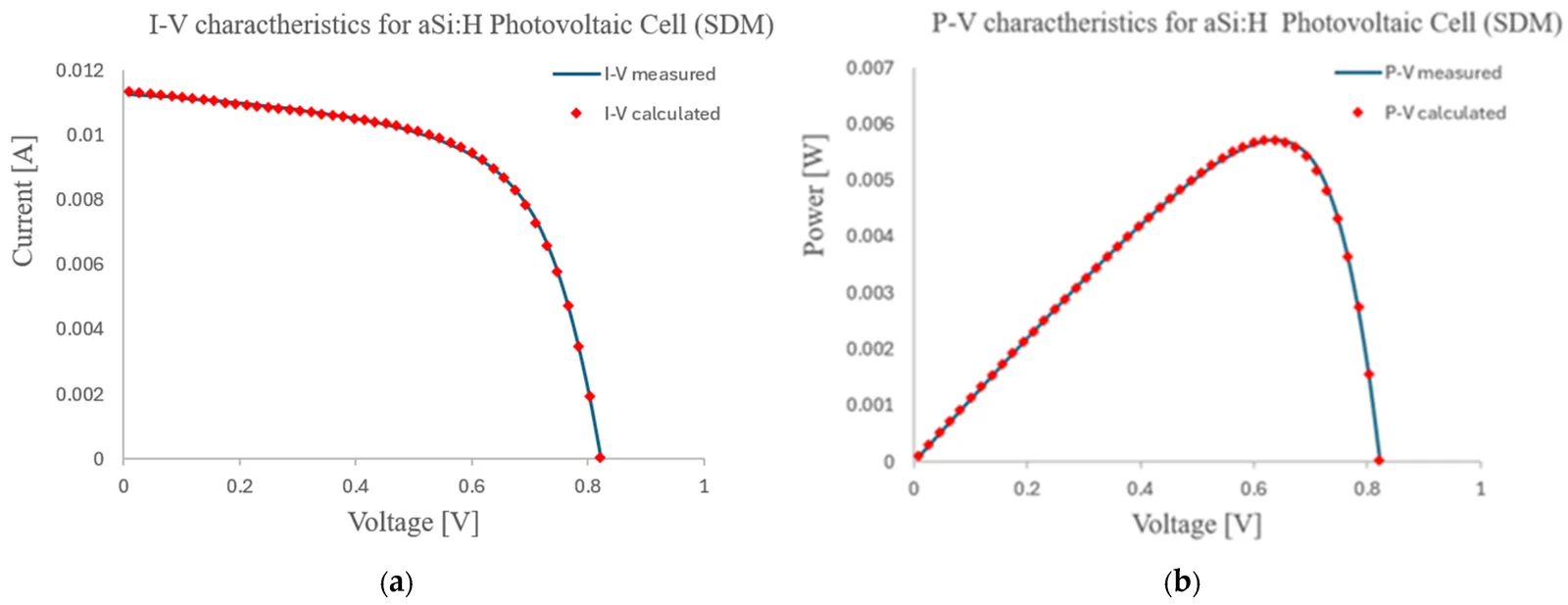
aSi:H photovoltaic cell characteristics measured vs. calculated—SDM: (**a**) I-V characteristic; (**b**) P-V characteristic.

**Figure 11:**
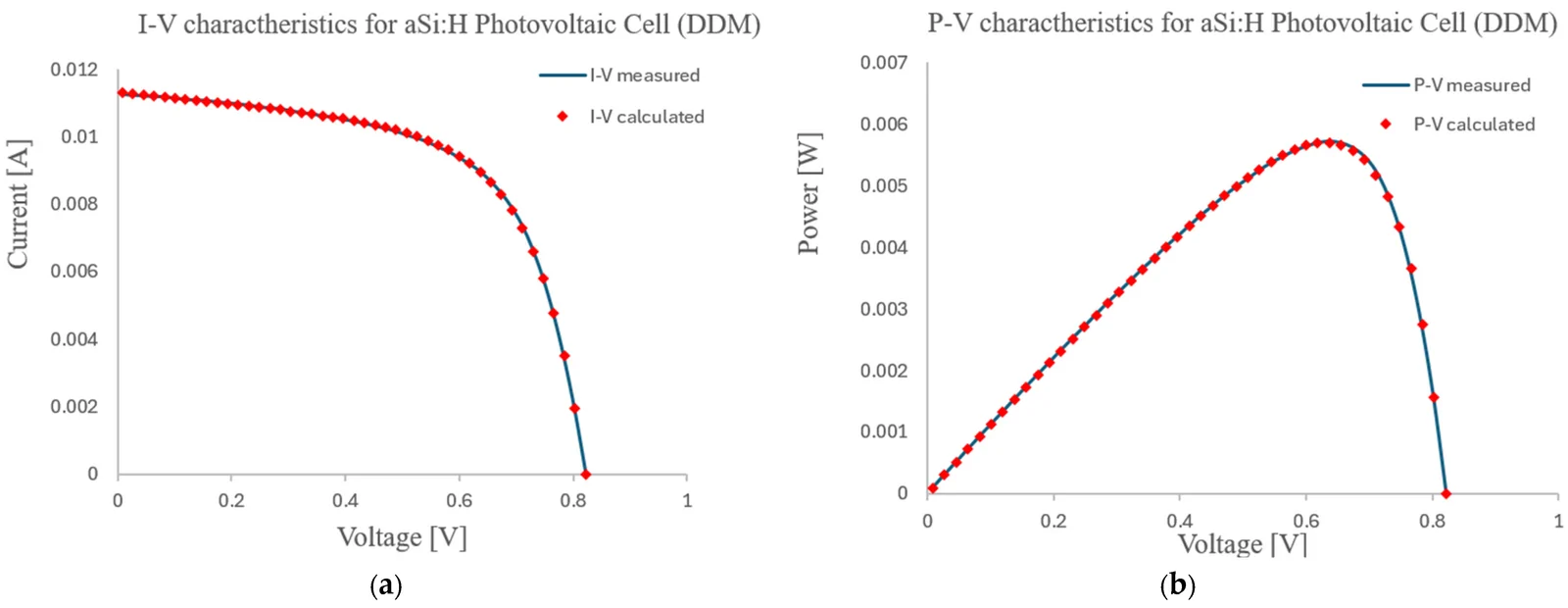
aSi:H photovoltaic cell characteristics measured vs. calculated—DDM: (**a**) I-V characteristic; (**b**) P-V characteristic.

**Figure 12:**
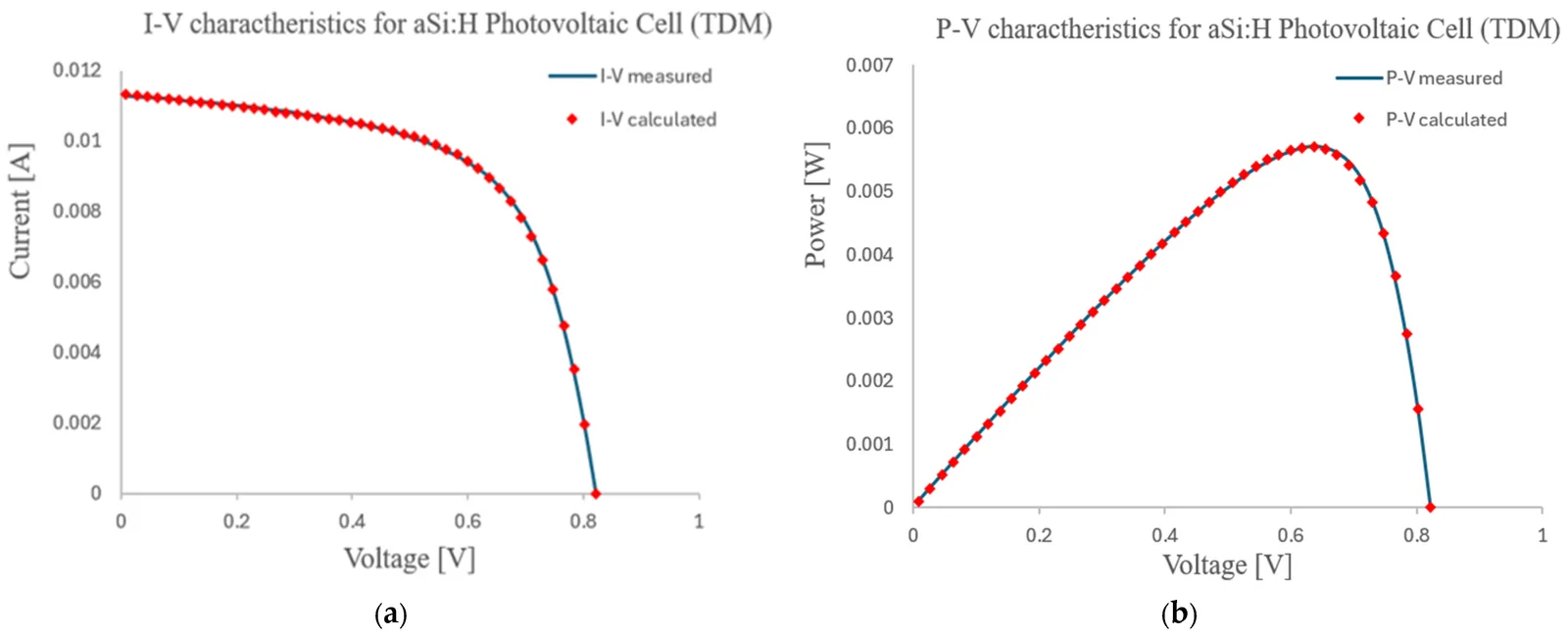
aSi:H photovoltaic cell characteristics measured vs. calculated—TDM: (**a**) I-V characteristic; (**b**) P-V characteristic.

The very good matching between the measured and calculated values using the CSA for the I-V and P-V characteristics are in Figure 10, in the case of SDM model, Figure 11 for DDM model, and Figure 12 for the TDM model. This proves an excellent estimation of the aSi:H parameters, confirmed also by Table 6.

**Figure 13:**
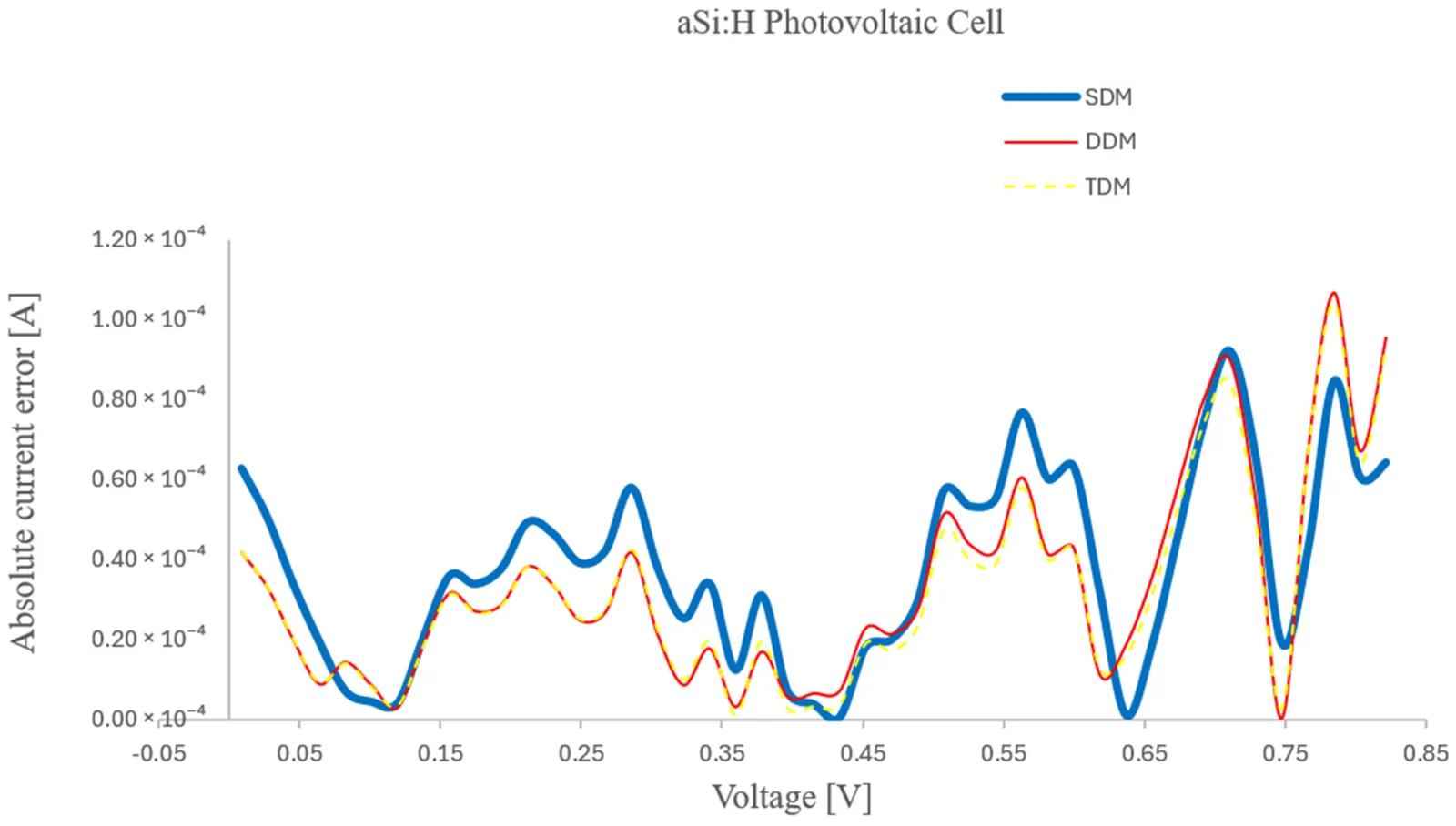
Absolute current error comparison for the three diode models for the aSi:H photovoltaic cell.

The absolute current errors for the three diode models are presented in Figure 13. The differences between the DDM and TDM models are relatively small. The absolute error for SDM model is higher for most voltages. DDM and TDM yield higher AE only at select voltages, particularly above 0.65 V. For a clearer picture, these values are detailed in the supplementary material in Table S4, which illustrates the measured voltage and current values, as well as the calculated current for the SDM, DDM, and TDM models.

**Figure 14:**
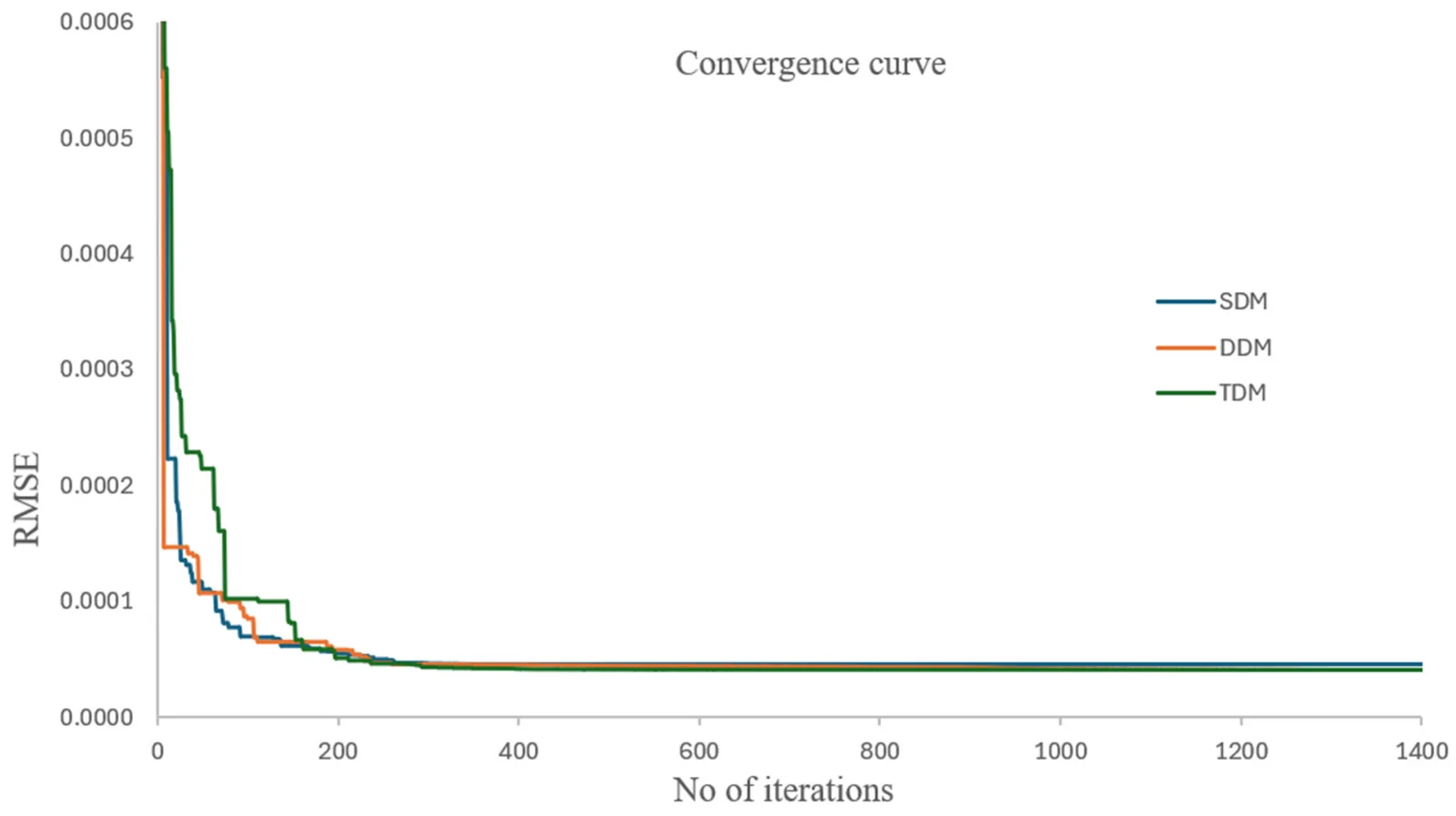
Convergence curves of CSA for the aSi:H photovoltaic cell.

Figure 14 shows the convergence curve for the CSA for the three models used in parameter extraction for the aSi:H photovoltaic cell. The SDM model has the best performance in terms of convergence speed, followed by the DDM model and then the TDM model.

A comparison between the CSA and several algorithms from the specialized literature is presented in Table 6. To make this comparison, six statistical metrics are used, and their values are calculated using the parameter values taken from the papers. RMSE obtained using CSA is the lowest or equal to the best one from the specialized literature for the SDM (equal) and DDM (the lowest) models. In the case of CSA, the coefficient of determination is also the highest among all models. This shows that the calculated and measured points match very well. For the TDM models, algorithms that do not respect the lower and upper limits specified in Table 1 were excluded.

The results for RMSE, MAE, MRE, MSE and R^2^ show that the CSA has the lowest or equal values in comparison with the other algorithms for the SDM and DDM models.

The values for the following RMSE metrics, such as mean, standard deviation, minimum, maximum, and interquartile range, are shown in Table 7. For the CSA, they are calculated after the algorithm runs 20 times. Because there are no results for these metrics in specialized literature, the comparison for the aSi:H photovoltaic cell is made only between the three models. The SDM model exhibits the lowest standard deviation, indicating a lower likelihood of extreme errors within an iteration class compared to the DDM and TDM models. The lowest mean is for the TDM model. Considering that this model has a lower standard deviation than the DDM model, it can be concluded that it is better to use the TDM model than the DDM model.

### 5.3. Photowatt-PWP201 Module

The I-V characteristic was measured at 45 °C, and 25 (V,I) pairs were considered. This module has 36 polycrystalline silicon photovoltaic cells connected in series. The dataset is most widely used for PV modules to verify the performance of the algorithms in parameter extraction.

The five, seven and nine parameters of the PWP201 photovoltaic module are presented in Table 8. These parameters are obtained using the CSA with 250 search agents and various iterations from 1000 to 2000 in increments of 250.

Extracting the parameters of a photovoltaic device is a multimodal problem. The objective function contains local minima where the algorithm can get trapped. If the global minimum is not reached, different parameter solutions may exist that yield very similar RMSE values. For example, for the TDM model with 1750 iterations, the RMSE value is 2.4250748 × 10^−3^—a value equal to the RMSE for 2000 iterations. The values of the reverse saturation currents and the ideality factor of diodes for the three mechanisms are very different; see Table 9.

Table S5 presents the distributions of the parameters of the PWP201 photovoltaic module across the 20 runs, which were summarized using the mean, median, standard deviation, minimum, maximum, interquartile range and coefficient of variation, utilizing the SDM, DDM, and TDM models. Only the results when 1750 iterations were applied, yielding the lowest RMSE value, are shown. IQR for all parameters is the lowest for the SDM model, which means the middle values are close together and more consistent. The highest are for the TDM model, so there is high variability. The analysis of the results in Table S5 for the SDM model reveals very low values for standard deviations, interquartile ranges (IQR), and coefficients of variation obtained from independent optimization runs. This demonstrates a high degree of stability and reproducibility in parameter estimation. The parameters appear very well-identified. In the case of the DDM model, *I_ph_, R_s_,* and *R_sh_* exhibit high stability, whereas *I_o_*_1_ and *I_o_*_2_ exhibit substantial variability. The ideality factors of diodes *n*_1_ and *n*_2_ have moderate variability, which indicates limited individual parameter identifiability. In the case of the TDM model, it does not provide the same level of identifiability for all parameters. *I_ph_*, *R_s_*, and *R_sh_* exhibit high stability, while the reverse saturation currents exhibit substantial variability, and the ideality factors of the diode have moderate variability.

**Figure 15:**
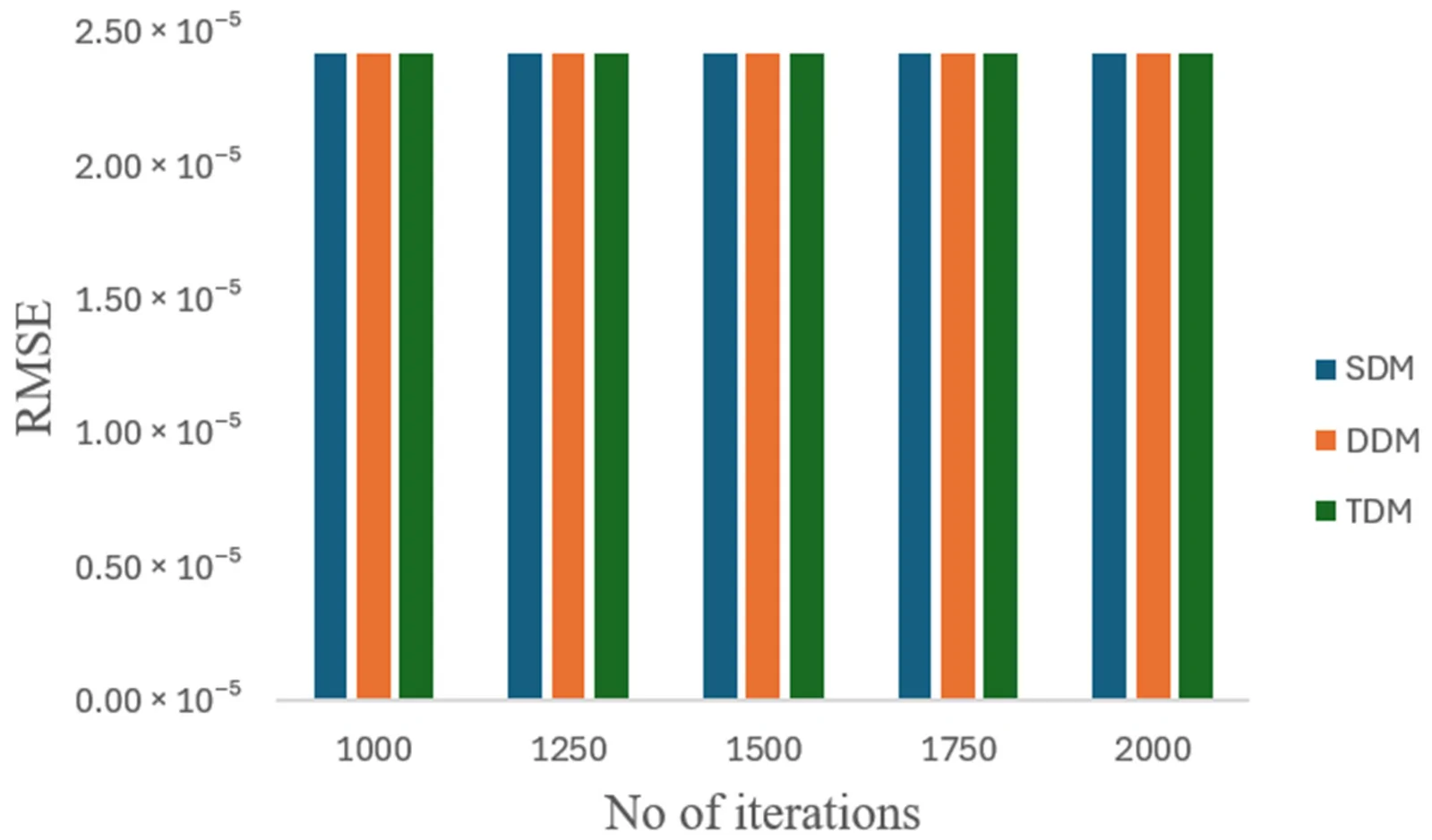
RMSE vs. iterations for the PWP201 photovoltaic module.

The most widely used statistical metric in analyzing parameter extraction is the RMSE. Figure 15 shows the variation in the RMSE for different numbers of iterations and the same number of populations, 250. There is a pattern, namely, if the number of iterations increases, the accuracy of determining the parameters remains almost constant. For the SDM model, the value of RMSE is 2.4250748 × 10^−3^. It is constant for increasing the iterations. In the case of DDM, it is the same, namely, 2.4250748 × 10^−3^. The lowest RMSE for the TDM model is 2.4250748 × 10^−3^. It was obtained for all iteration classes, except 1250 iterations. The conclusion of the study of variation in the number of iterations is that the algorithm provides very good results from 1750 iterations for all three models.

**Figure 16:**
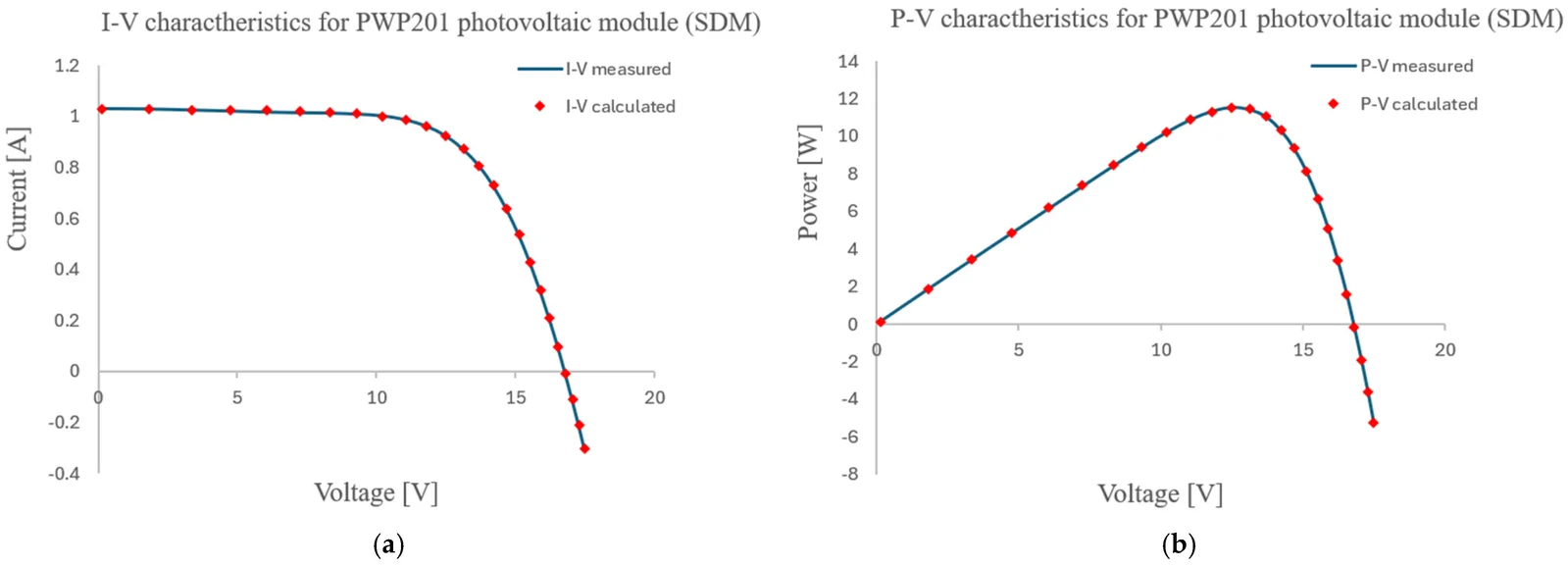
PWP201 photovoltaic module characteristics measured vs. calculated—SDM: (**a**) I-V characteristic; (**b**) P-V characteristic.

**Figure 17:**
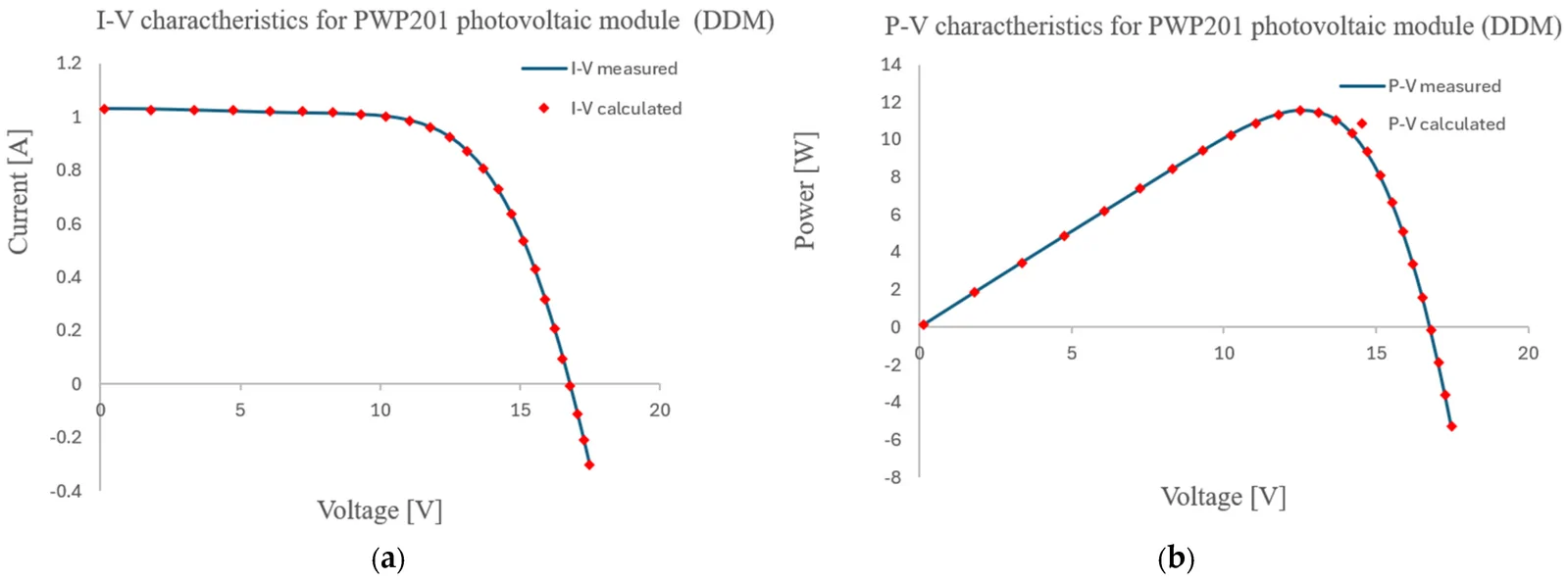
PWP201 photovoltaic module characteristics measured vs. calculated—DDM: (**a**) I-V characteristic; (**b**) P-V characteristic.

**Figure 18:**
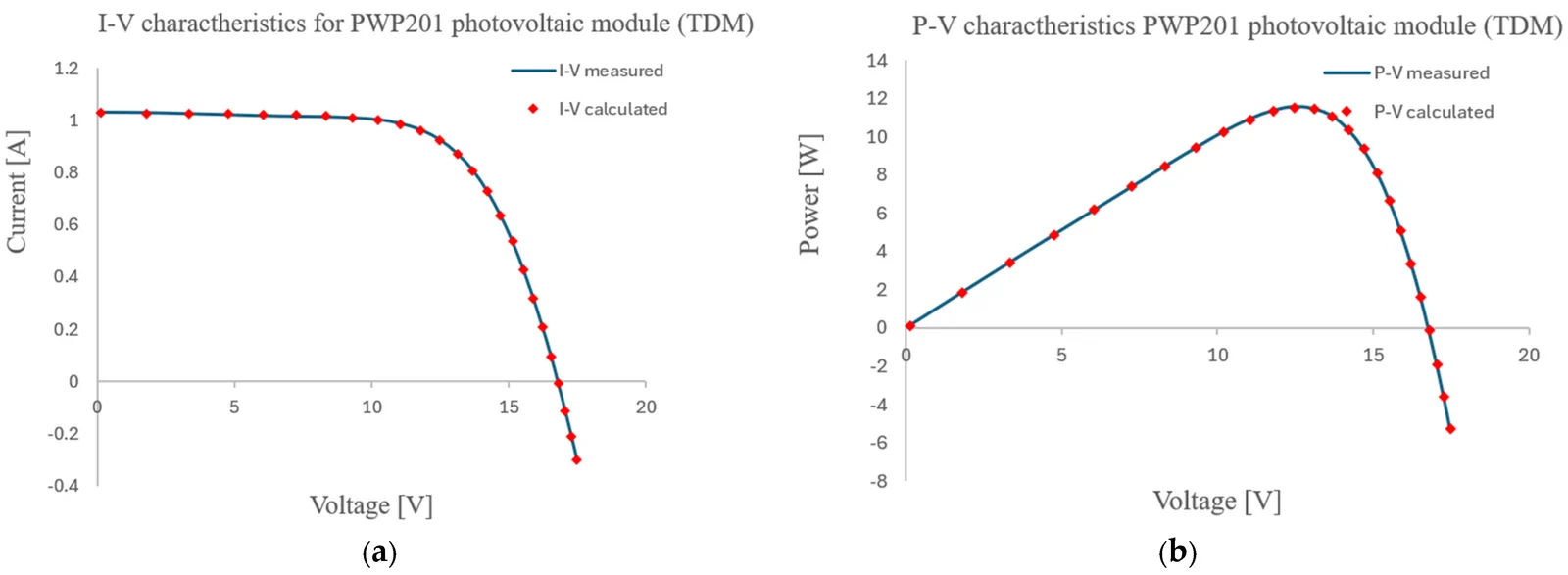
PWP201 photovoltaic module characteristics measured vs. calculated—TDM: (**a**) I-V characteristic; (**b**) P-V characteristic.

The very good matching between the measured and calculated values using the CSA for the I-V and P-V characteristics is illustrated in Figure 16 in the case of the SDM model, Figure 17 for the DDM model, and Figure 18 for the TDM model. These results confirm an excellent estimation of the PWP201 photovoltaic module, as further supported by Table 7.

**Figure 19:**
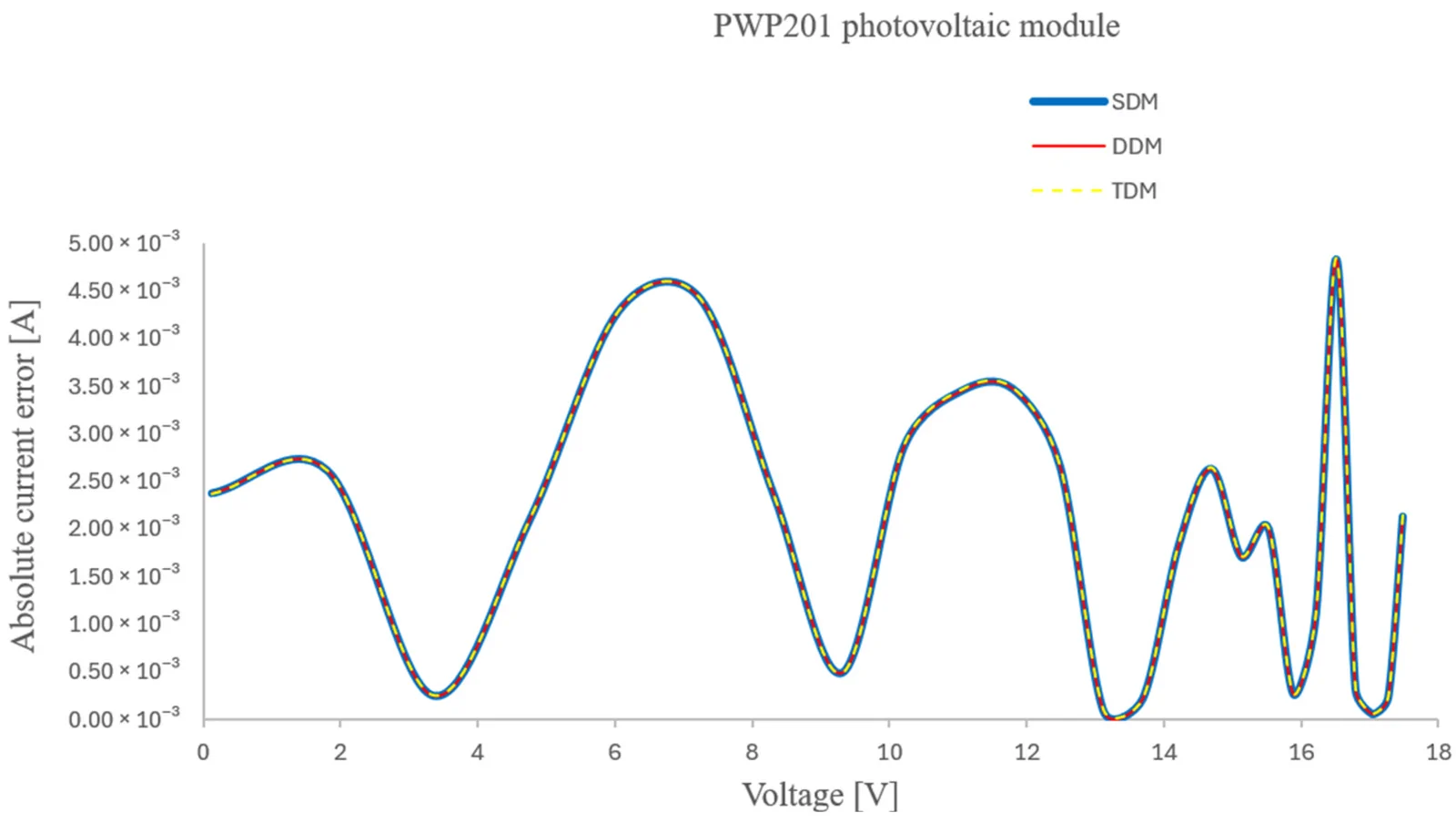
Absolute current error comparison for the three diode models for the PWP201 photovoltaic module.

The absolute current errors for the three diode models are presented in Figure 19. The differences between the three models, SDM, DDM and TDM, are very small. There are minima and maxima. The highest values for the absolute current error occur at 7 V and 16.5 V. For better visualization, these values are presented in the supplementary files in Table S6, which contains the measured voltage and current values, as well as the calculated current for the SDM, DDM, and TDM models.

**Figure 20:**
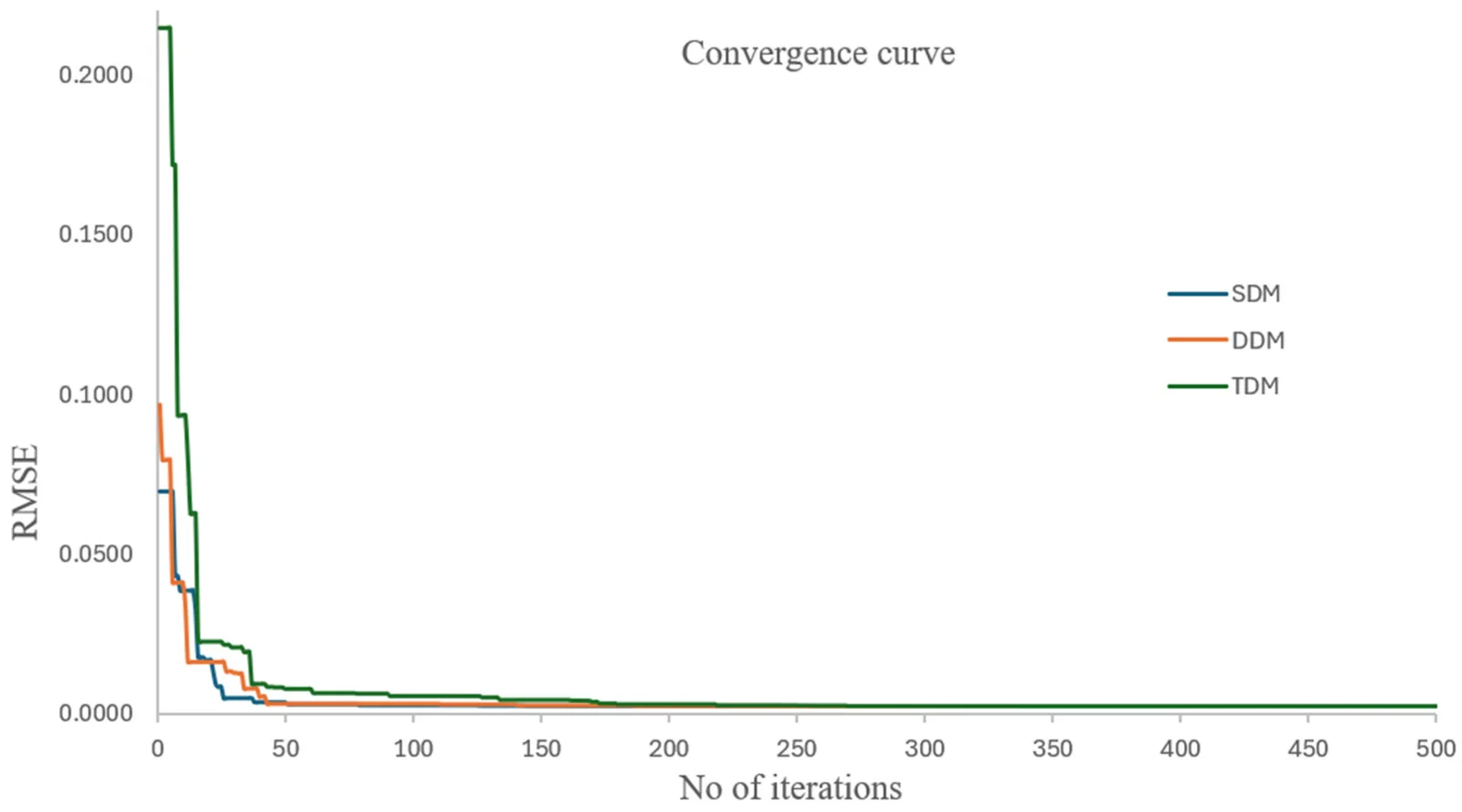
Convergence curves of CSA for the PWP201 photovoltaic module.

Figure 20 shows the convergence curve for the CSA for the three models used in parameter extraction for the PWP201 photovoltaic module. The SDM model has the best performance in terms of convergence speed, followed by the DDM model and then the TDM model.

A comparison between the CSA and several algorithms from the specialized literature is presented in Table 9. RMSE of the obCSACSA algorithm is the lowest or equal to the best one from the specialized literature for the SDM (equal) and DDM (the lowest) model. RMSE for the TDM model obtained using CSA is slightly lower than that obtained using HSDA. In the case of CSA, the coefficient of determination is also the highest among all models. This shows that the calculated and measured points match very well.

The values for the following metrics of the RMSE, such as mean, standard deviation, minimum, maximum, and interquartile range, are shown in Table 10. For the CSA, they are calculated after the algorithm runs 20 times. The results for the other algorithms are from specialized literature; the comparison has some limitations due to differences in population sizes, iteration counts, stopping criteria, and numbers of runs for the references. In the case of the SDM model, the mean, standard deviation, minimum, and maximum of RMSE are better than those of the other algorithms considered. For FDB-SFS and CSAO, the mean, minimum, and maximum are the same as CSA, but the standard deviation is three times higher than that for CSA and more than two times higher, respectively, which means the CSA is more robust. For the other algorithms, all metrics have the worst values. So, the CSA has the best performance. In the case of the DDM model and TDM, CSA is compared with STF. In the case of the DDM model, CSA has the lowest standard deviation, which means it is more robust than STF, but in the case of the TDM model, the roles are reversed. The IQR increases from the SDM model to the TDM model; the highest is for the TDM model, so there is high variability for the TDM model.

## 6. Computing Time

Computation time for extracting photovoltaic cell and module parameters is critical, particularly in industrial manufacturing. As it depends on the computer’s specifications to run the algorithm, it is crucial to have the features of the computers and the software used for a fair comparison. The hardware configuration used to run algorithms was mentioned above.

Table 11 shows the necessary computing time function of the type of photovoltaic device, model used, and the iterations. As expected, increasing the number of iterations across each PV device and diode model leads to a corresponding increase in computation time. Increasing the iteration count from 1000 to 2000 results in a greater than 90% increase in computation time. In our case, this increase, even in percentage, is high, around 100%; in fact, the growth is around 1 s or less in general, but the accuracy in parameter extraction increases considerably, as can be seen from Table 2, Table 5 and Table 8. Increasing the number of point pairs in the dataset does not automatically increase or decrease the computation time, as it is mentioned before, as for RTC there are 26 pairs, for aSi:H, 46 pairs, and for PWP201, 25 pairs.

Transitioning from the SDM to the DDM and subsequently to the TDM resulted in increased computation time. In the case of 1750 iterations for RTC, the computing time increases from 1.45 s to 2.02 s and 2.35 s; for aSi:H from 1.37 s to 1.61 s and 2.18 s; and for PWP201 from 1.58 to 1.59 and 1.88 s.

As previously mentioned, for RTC there are 26 pairs, for aSi:H, 46 pairs, and for PWP201, 25 pairs, and the computing time oscillates for all models. If the SDM model is applied, the computing time for aSi:H is lower than for RTC for 1000, 1250, and 1750, and higher for the other two. The computation time for PWP201 is lower than for aSi:H in the case of the first three and higher for the last two. The conclusion is that no pattern can be established.

**Figure 21:**
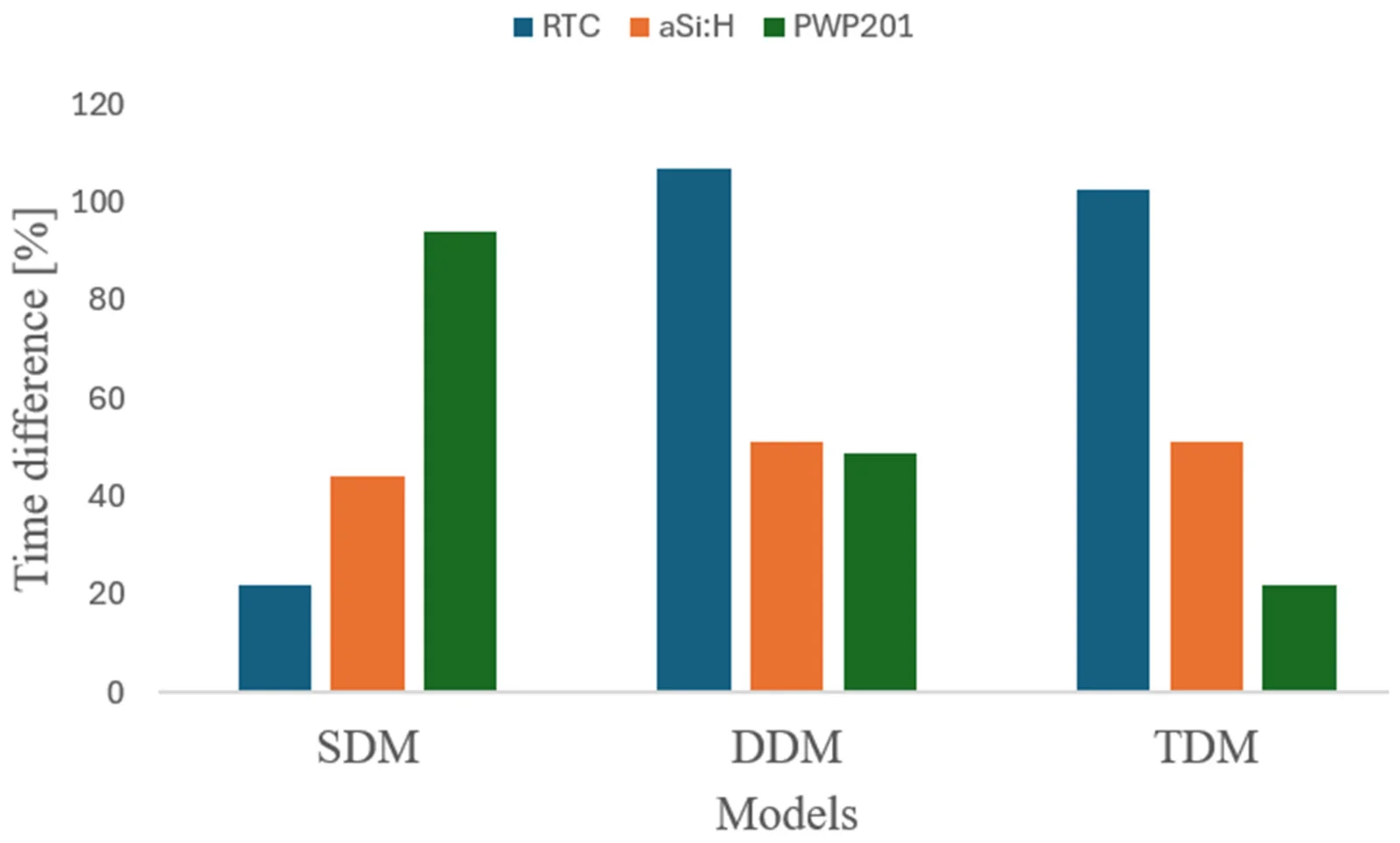
Computing time difference.

Figure 21 shows the time difference in percentage between the computing time for running CSA in the case of 1000 iterations, where the accuracy of the RMSE was acceptable, and 1750 iterations which offer the best RMSE for almost all cases. The computing time behavior differs again among the three PV devices. In the case of RTC, the time difference increases significantly from the SDM to the DDM model and remains almost the same for TDM. In the case of aSi:H, this has almost the same behavior as in the RTC case, but the increase is much smaller. PWP201 exhibits the opposite behavior. The difference decreases significantly from the SDM to the TDM model.

In the case of BWOA, the specifications are the same as for CSA. The computing time is significantly shorter for CSA in comparison with BWOA; for the SDM model, it is 1.45023 s for CSA (250 search agents and 1750 iterations) and 2.88978 s for BWOA (175 search agents and 1500 iterations), and 2.029755 s for CSA and 2.1669 s for BWOA (250 search agents and 1500 iterations). The same difference is also observed for the other PV devices considered. For the SDM model, the computing time is 1.9598 s (200 search agents and 1500 iterations) for aSi:H and 2.3 s for PWP201 in the BWOA case.

## 7. Conclusions

The CSA was proposed and adapted to extract the five-SDM model, seven-DDM model and nine-parameter-TDM model of the RTC and aSi:H photovoltaic cells and PWP201 photovoltaic module. The parameters were extracted for 250 search agents and a different number of iterations, from 1000 to 2000 with a step of 250. The best results are obtained for 250 search agents and 1750 iterations in almost all cases except one.

Six statistical metrics, such as RMSE, MAE, MBE, MRE, MSE, and R^2^ are calculated to verify the performance of the CSA. Furthermore, the mean, standard deviation, minimum, maximum, interquartile range and coefficient of variation are used to prove the robustness and stability of the CSA. The analysis shows that, in the case of RMSE and using the SDM model, CSA gives equal values for RTC, aSi:H, and PWP 201, and in the case of the TDM model for PWM 201, it matches the best ones from specialized literature. In the rest, the RMSE calculated with CSA is lower. This proves that the CSA is competitive in identifying the parameters of photovoltaic cells and modules, while also being robust, stable, and reliable.

Computation time was analyzed as a function of the three photovoltaic devices, models, and iteration count. The analysis shows that the growth of the iterations led to increasing computing time, in all but two cases. Transitioning from the SDM to the DDM and TDM models increased computation time, though the rate of increase varies significantly.

The I-V and P-V characteristics show an overlap between the measured values and the calculated ones over the entire voltage interval. To provide a complete picture, the absolute current error is calculated and presented in the supplementary tables for all three photovoltaic devices.

The high accuracy of parameter extraction combined with low computation time makes the CSA an effective tool for both research and industrial applications.

This study has some limitations, such as being conducted under a single irradiation and temperature, only 250 search agents being used, 20 runs, and the competing methods were evaluated under different settings in most cases (population sizes, iteration counts, stopping criteria, and numbers of runs). Future work will address all these limitations. A controlled comparison under a common function-evaluation budget and identical stopping criteria, with multiple independent runs for all methods, is an important direction for future work. CSA will be applied to datasets obtained for different temperatures and irradiations. The number of search agents will be varied to determine its impact on accuracy and computation time. Hybridization could be a solution to improve the performance of the CSA for the TDM model.

## Figures and Tables

**Table 1:** Boundaries for the photovoltaic cells and modules under test.

Device Under Test	Boundaries	I_ph_ [A]	I_o,1,2,3_ [A]	n_1,2,3_	R_s_ [Ω]	R_sh_ [Ω]
RTC France	LB	0	10^−12^	1	0	0
	UB	1	10^−5^	2.5	0.5	100
aSi:H	LB	0	10^−12^	1	0	0
	UB	0.1	10^−5^	5	0.5	1000
PWP201	LB	0	10^−12^	1	0	0
	UB	2	10^−5^	2	2	1000

**Table 2:** Extracted parameters of the RTC photovoltaic cell.

Diode Models	No. of Interations	I_ph_ [A]	I_o1_ [A]	n_1_	R_s_ [Ω]	R_sh_ [Ω]	I_o2_ [A]	n_2_	I_o2_ [A]	n_3_
SDM	1000	0.76077763	3.2556918 × 10^−7^	1.4819736	0.03634814	53.956095				
	1250	0.76077553	3.2302080 × 10^−7^	1.4811836	0.03637709	53.718526				
	1500	0.76077553	3.2302081 × 10^−7^	1.4811836	0.03637709	53.718521				
	1750	0.76077553	3.2302080 × 10^−7^	1.4811836	0.03637709	53.718524				
	2000	0.76077553	3.2302079 × 10^−7^	1.4811836	0.03637709	53.718522				
DDM	1000	0.76078610	1.7582849 × 10^−8^	1.7844631	0.03639918	53.563523	3.1603889 × 10^−7^	1.4794361		
	1250	0.76083352	2.9195457 × 10^−7^	1.4721686	0.03646654	56.125565	1.5192976 × 10^−6^	2.4999597		
	1500	0.76086120	2.9069669 × 10^−7^	1.4714653	0.03655169	53.124189	7.9825154 × 10^−7^	2.4127050		
	1750	0.76080383	2.7373741 × 10^−7^	1.4663527	0.03654585	55.928814	1.8921077 × 10^−6^	2.4506855		
	2000	0.76077673	2.2999983 × 10^−7^	1.4505100	0.03687678	57.787820	3.5661149 × 10^−6^	2.4774657		
TDM	1000	0.76077371	3.2030170 × 10^−7^	1.4804343	0.03638603	53.850668	2.7294322 × 10^−8^	2.1797347	3.5839729 × 10^−8^	2.4847713
	1250	0.76078835	2.7954928 × 10^−7^	1.4681390	0.03658253	54.817096	4.8743140 × 10^−7^	2.2525375	5.8144593 × 10^−7^	2.4987341
	1500	0.76077840	1.4024686 × 10^−8^	1.8047508	0.03663265	55.256432	1.5483203 × 10^−6^	2.4723362	2.7303894 × 10^−7^	1.4659925
	1750	0.76077712	2.7964371 × 10^−7^	1.4687414	0.03654850	54.714902	3.8697517 × 10^−9^	1.5372879	1.1507836 × 10^−6^	2.3980241
	2000	0.76078422	2.5333125 × 10^−7^	1.4592842	0.03670956	56.273168	1.2699642 × 10^−6^	2.3691766	9.3118388 × 10^−7^	2.4983011

**Table 4:** RMSE result statistics for the three models, SDM, DDM and TDM, of the RTC photovoltaic cell.

Models	Algorithm	Mean	Str Dev	Min	Max	IQR
SDM	**CSA**	**9.8602188** × **10^−4^**	**2.131462** × **10^−17^**	**9.8602188** × **10^−4^**	**9.8602188** × **10^−4^**	**2.1923980** × **10^−17^**
	IAPO	9.8602188 × 10^−4^	2.132698 × 10^−17^	9.8602188 × 10^−4^	9.8602188 × 10^−4^	-
	SJAYA	9.860219 × 10^−4^	2.132116 × 10^−17^	9.860219 × 10^−4^	9.860219 × 10^−4^	-
	IHGS [40]	9.86022 × 10^−4^	5.8351 × 10^−12^	9.86022 × 10^−4^	9.86022 × 10^−4^	
	WaOA	9.89112 × 10^−4^	2.775 × 10^−17^	9.89085 × 10^−4^	9.89231 × 10^−4^	-
	CSAO	9.8602188 × 10^−4^	4.912440 × 10^−17^	9.8602188 × 10^−4^	9.8602188 × 10^−4^	-
	FDB-SFS	1.03 × 10^−3^	4.86 × 10^−5^	9.86023 × 10^−4^	1.17 × 10^−3^	-
	I-CPA	4.4400 × 10^−3^	4.2832 × 10^−3^	9.9862 × 10^−4^	1.8243 × 10^−2^	-
	EINFO	10.2301 × 10^−4^	4.5174 × 10^−17^	10.2301 × 10^−4^	10.2301 × 10^−4^	
	AGDE	3.93 × 10^−3^	1.08 × 10^−3^	2.19 × 10^−3^	6.53 × 10^−3^	-
	SFOA	4.06978 × 10^−3^	8.1306 × 10^−3^	3.9843 × 10^−3^	5.5984 × 10^−3^	-
	SPBO	4.59 × 10^−3^	8.99 × 10^−2^	9.93 × 10^−4^	2.22 × 10^−1^	-
DDM	CSA	**9.8914923** × **10^−4^**	**1.6880171** × **10^−6^**	**9.7670008** × **10^−4^**	**1.0544886** × **10^−3^**	**2.5683721** × **10^−6^**
	IAPO	9.8339687 × 10^−4^	1.7536 × 10^−6^	9.82484851 × 10^−4^	9.8887307 × 10^−4^	-
	CSAO	9.8248488 × 10^−4^	1.505925 × 10^−6^	9.8248488 × 10^−4^	9.8700481 × 10^−4^	
	SJAYA	9.849674 × 10^−4^	1.1640 × 10^−6^	9.82633 × 10^−4^	9.86986 × 10^−4^	-
	IHGS [40]	9.8408 × 10^−4^	1.9832 × 10^−6^	9.8408 × 10^−4^	9.8408 × 10^−4^	-
	EINFO	9.8431 × 10^−4^	2.0087 × 10^−6^	9.8431 × 10^−4^	9.8431 × 10^−4^	-
	I-CPA	4.3539 × 10^−3^	3.0441 × 10^−3^	1.0252 × 10^−3^	1.2067 × 10^−2^	-
TDM	**CSA**	**9.820852** × **10^−4^**	**1.3161084** × **10^−6^**	**9.7805717** × **10^−4^**	**9.8975427** × **10^−4^**	**5.3280702** × **10^−8^**
	CSAO	9.829718 × 10^−4^	1.5108249 × 10^−6^	9.8248488 × 10^−4^	9.895424 × 10^−4^	-
	*μ*AFCSO [36]	1.4196 × 10^−3^	4.5925 × 10^−4^	9.8295 × 10^−4^	3.5422 × 10^−3^	-

**Table 5:** Extracted parameters of the aSi:H photovoltaic cell.

Diode Models	No. of Iterations	I_ph_ [A]	I_o1_ [A]	n_1_	R_s_ [Ω]	R_sh_ [Ω]	I_o2_ [A]	n_2_	I_o2_ [A]	n_3_
SDM	1000	0.01134472	7.3493728 × 10^−7^	3.3684180	8.979752 × 10^−14^	523.15342				
	1250	0.01134472	7.3493727 × 10^−7^	3.3684180	9.055559 × 10^−14^	523.15342				
	1500	0.01134472	7.3493727 × 10^−7^	3.3684180	1.859501 × 10^−14^	523.15342				
	1750	0.01134472	7.3493728 × 10^−7^	3.3684180	9.095173 × 10^−14^	523.15342				
	2000	0.01134472	7.3493726 × 10^−7^	3.3684180	1.832666 × 10^−14^	523.15342				
DDM	1000	0.011330952	1.4693614 × 10^−7^	2.9574814	0.14326309	575.9996	3.9787853 × 10^−6^	4.9283668		
	1250	0.011337743	5.4672348 × 10^−6^	2.7268433	0.49990482	588.9127	5.2382303 × 10^−6^	4.9901569		
	1500	0.011331864	5.5192865 × 10^−6^	4.9999971	0.49875884	600.6187	4.9809881 × 10^−8^	2.7090407		
	1750	0.011330184	5.5421610 × 10^−6^	4.9999864	0.49787087	603.4121	5.0078614 × 10^−8^	2.7106444		
	2000	0.011332932	5.4218914 × 10^−6^	4.9991216	0.49979385	597.8611	5.2444014 × 10^−8^	2.7192315		
TDM	1000	0.01134215	4.9526248 × 10^−6^	4.9901109	0.49978880	579.20414	6.3662813 × 10^−8^	2.7575369	3.1902638 × 10^−9^	4.8240478
	1250	0.01133579	3.3492080 × 10^−12^	3.4677610	0.49999977	584.51909	7.4893035 × 10^−8^	2.7935530	4.6927577 × 10^−6^	4.9716760
	1500	0.01133210	1.7269040 × 10^−8^	4.9997667	0.47499038	598.23759	5.4689862 × 10^−8^	2.7287464	5.4004644 × 10^−6^	5.0000000
	1750	0.01133174	6.3896666 × 10^−7^	5.0000000	0.50000000	600.66725	4.8684611 × 10^−6^	5.0000000	5.0261039 × 10^−8^	2.7108864
	2000	0.01133174	1.0000000 × 10^−12^	3.6924616	0.50000000	600.66722	5.0260965 × 10^−8^	2.7108861	5.5074241 × 10^−6^	5.0000000

**Table 6:** Values of the statistical test for the aSi:H photovoltaic cell.

Models	Algoritms	RMSE [10^−5^]	MAE [10^−5^]	MBE	MRE	MSE	R^2^
SDM	**CSA**	**4.61232**	**3.94016**	**1.22294** × **10^−12^**	**1.95346** × **10^−2^**	**2.12735** × **10^−9^**	**0.9996661**
	BWOA [41]	4.61232	3.94538	2.15906 × 10^−12^	2161571 × 10^−2^	2.12996 × 10^−9^	0.9996657
	ISOA	4.61276	3.94016	−1.65429 × 10^−12^	1.95346 × 10^−2^	2.12735 × 10^−9^	0.9996661
	HSDA	4.61945	3.96462	4.1353 × 10^−7^	1.97802 × 10^−2^	2.13394 × 10^−9^	0.9996651
	BMOA [42]	8.2115	6.9524	−1.6201 × 10^−6^	7.3431 × 10^−2^	6.7430 × 10^−9^	0.9995478
	5P [43]	20.8631	18.995	−1.29276 × 10^−5^	1.2441 × 10^−2^	4.3526 × 10^−8^	0.993170
DDM	**CSA**	**4.095901**	**3.29722**	**−6.29667** × **10^−10^**	**2.6270** × **10^−3^**	**1.67764** × **10^−9^**	**0.999736**
	BWOA	4.25861	3.46538	4.33325 × 10^−9^	7.87608 × 10^−3^	2.12996 × 10^−9^	0.999715
	ISOA	4.25963	3.57289	8.70312 × 10^−8^	6.2511 × 10^−3^	1.81445 × 10^−9^	0.999715
	HSDA	4.49735	3.63262	2.63015 × 10^−8^	8.01231 × 10^−3^	2.13394 × 10^−9^	0.9996651
	BMOA	5.3511	3.76321	3.7606 × 10^−7^	4.6531 × 10^−3^	6.7430 × 10^−9^	0.999467
TDM	**CSA**	**4.094393**	**3.28847**	**2.71235** × **10^−12^**	**2.5674** × **10^−3^**	**1.6764** × **10^−9^**	**0.999736**

**Table 7:** RMSE result statistics for the three models, SDM, DDM and TDM, of the aSi:H photovoltaic cell.

Models	Algorithm	Mean	Str Dev	Min	Max	IQR
SDM	CSA	4.6123219 × 10^−5^	8.0358481 × 10^−20^	4.6123219 × 10^−5^	4.6123219 × 10^−5^	7.4538899 × 10^−20^
DDM	CSA	4.2997847 × 10^−5^	1.1911673 × 10^−6^	4.0949367 × 10^−5^	4.5837338 × 10^−5^	8.4678395 × 10^−7^
TDM	CSA	4.2143800 × 10^−5^	9.6590522 × 10^−7^	4.0943938 × 10^−5^	4.4185988 × 10^−5^	1.7901463 × 10^−6^

**Table 8:** Extracted parameters of the PWP201 photovoltaic module.

Diode Models	No. of Iterations	I_ph_ [A]	I_o1_ [A]	n_1_	R_s_ [Ω]	R_sh_ [Ω]	I_o2_ [A]	n_2_	I_o2_ [A]	n_3_
SDM	1000	1.0305143	3.4822628 × 10^−6^	1.351190	1.201271	981.98224				
	1250	1.0305143	3.4822629 × 10^−6^	1.351190	1.201271	981.98229				
	1500	1.0305143	3.4822629 × 10^−6^	1.351190	1.201271	981.98223				
	1750	1.0305143	3.4822627 × 10^−6^	1.351190	1.201271	981.98218				
	2000	1.0305143	3.4822629 × 10^−6^	1.351190	1.201271	981.98224				
DDM	1000	1.0305143	1.0000000 × 10^−12^	1.443541	1.201271	981.98264	3.4822627 × 10^−6^	1.35118986		
	1250	1.0305143	1.0000002 × 10^−12^	1.981751	1.201271	981.98233	3.4822627 × 10^−6^	1.35118986		
	1500	1.0305143	1.0668199 × 10^−9^	1.351189	1.201271	981.98224	3.4811961 × 10^−6^	1.35118986		
	1750	1.0305143	1.0000000 × 10^−12^	2.000000	1.201271	981.98233	3.4822630 × 10^−6^	1.35118986		
	2000	1.0305143	1.9877393 × 10^−6^	1.351189	1.201272	981.97934	1.4945047 × 10^−6^	1.35118944		
TDM	1000	1.0305143	1.0000002 × 10^−12^	1.848263	1.201271	981.98241	3.4822627 × 10^−6^	1.35118986	1.0000001 × 10^−12^	1.764106
	1250	1.0304448	1.5229610 × 10^−12^	1.711698	1.200569	993.14675	1.0000000 × 10^−12^	1.28449928	3.5055482 × 10^−6^	1.351895
	1500	1.0305147	3.3055253 × 10^−6^	1.351187	1.201273	981.92586	1.7664643 × 10^−7^	1.35119733	1.0000000 × 10^−12^	1.943178
	1750	1.0305143	1.0000000 × 10^−12^	1.874633	1.201271	981.98696	2.4900842 × 10^−8^	1.35118303	3.4573642 × 10^−6^	1.35119
	2000	1.0305143	3.3015917 × 10^−6^	1.351190	1.201271	981.98228	1.8067009 × 10^−7^	1.35119000	1.0000001 × 10^−12^	1.988663

**Table 9:** Values of the statistical test for the PWP201 photovoltaic module.

Models	Algoritms	RMSE [10^−3^]	MAE [10^−3^]	MBE	MRE	MSE	R^2^
SDM	**CSA**	**2.4250748**	**1.95695**	**4.25074** × **10^−9^**	**5.9966** × **10^−3^**	**5.88099** × **10^−6^**	**0.9999701**
	BWOA [41]	2.4250748	1.95695	−1.66145 × 10^−10^	5.99652 × 10^−3^	5.88098 × 10^−6^	0.9999701
	I-CPA	2.4374465	1.95711	−5.15472 × 10^−6^	4.99686 × 10^−3^	5.94115 × 10^−6^	0.9999698
	CSAO	2.4250935	1.95733	−5.89052 × 10^−6^	5.91257 × 10^−3^	5.88108 × 10^−6^	0.9999701
	SJAYA	2.4250885	1.9566	7.27024 × 10^−6^	6.05944 × 10^−3^	5.88105 × 10^−6^	0.9999701
	HSDA	2.4250748	1.95695	−9.49346 × 10^−6^	5.99652 × 10^−3^	5.88099 × 10^−6^	0.9999701
	En-PDO	2.5988311	2.13962	4.48403 × 10^−6^	1.17348 × 10^−2^	6.75392 × 10^−6^	0.9999656
	ISCE	2.425075	1.95696	−9.49475 × 10^−10^	5.99652 × 10^−3^	5.88099 × 10^−6^	0.9999701
	EINFO	2.6049489	2.13377	8.19826 × 10^−5^	1.22547 × 10^−2^	6.78576 × 10^−6^	0.9999655
	WDOWOAPSO [44]	2.63133	2.1489	−3.64647 × 10^−5^	3.2095 × 10^−1^	6.92121 × 10^−6^	0.9999649
	GCPSO [45]	2.63132	2.149	−3.67245 × 10^−5^	3.2156 × 10^−1^	6.92121 × 10^−6^	0.9999648
	BMOA [42]	2.6377889	2.16724	−3.19738 × 10^−6^	8.26361 × 10^−3^	6.95793 × 10^−6^	0.9999646
	5P [43]	4.1251	3.33409	1.80911 × 10^−3^	1.97806 × 10^−2^	4.04863 × 10^−8^	0.9986521
DDM	**CSA**	**2.4250748**	**1.95695**	**4.25074** × **10^−9^**	**5.9966** × **10^−3^**	**5.88099** × **10^−6^**	**0.9999701**
	BWOA	2.42601	1.95928	−5.10197 × 10^−6^	5.92945 × 10^−3^	5.88551 × 10^−6^	0.9999700
	HSDA	2.587754	2.14548	−5.3062 × 10^−5^	2.8495 × 10^−3^	1.2221 × 10^−5^	0.999966
	AEO [46]	2.606962	2.12558	4.41713 × 10^−7^	2.8541 × 10^−3^	1.2223 × 10^−5^	0.999965
	WDOWOAPSO [44]	2.631325	214891	−3.64546 × 10^−5^	1.37405 × 10^−2^	1.2324 × 10^−5^	0.999965
	GCPSO [45]	2.6313	214891	−3.64547 × 10^−5^	1.37405 × 10^−2^	1.2324 × 10^−5^	0.999965
	BMOA	4.1767	3.6456	5.3913 × 10^−4^	5.6796 × 10^−3^	1.7445 × 10^−6^	0.999467
TDM	**CSA**	**2.4250748**	**1.956621**	**6.60279** × **10^−8^**	**6.0211** × **10^−2^**	**5.88102** × **10^−6^**	**0.9999701**
	HSDA	2.425075	1.95707	1.04708 × **10^−8^**	6.0325 × 10^−3^	5.88099 × **10^−6^**	0.9999701
	STF [47]	2.425083	1.95667	4.06957 × **10^−8^**	6.0812 × 10^−3^	5.88103 × **10^−6^**	**0.9999701**
	OBEDO [48]	2.437738	1.99952	−1.56111 × **10^−4^**	5.14526 × 10^−3^	5.94257 × **10^−6^**	**0.9999698**
	TGA	3.72591	2.34256	−2.23123 × **10^−4^**	6.23145 × 10^−3^	6.52147 × **10^−6^**	**0.9999676**

**Table 10:** RMSE result statistics for the three models, SDM, DDM and TDM, of the PWP201 photovoltaic module.

Models	Algorithm	Mean	Str Dev	Min	Max	IQR
SDM	**CSA**	**2.4250748** × **10^−3^**	**1.4202809** × **10^−17^**	**2.4250748** × **10^−3^**	**2.4250748** × **10^−3^**	**1.4202809** × **10^−17^**
	FDB-SFS	2.42*10^−3^	5.24 × 10^−17^	2.42 × 10^−3^	2.42 × 10^−3^	-
	CSAO	2.4250748 × 10^−3^	3.54454698 × 10^−17^	2.4250748 × 10^−3^	2.4250748 × 10^−3^	-
	SJAYA	2.431177 × 10^−3^	3.342175 × 10^−5^	2.425075 × 10^−3^	2.608134 × 10^−3^	-
	GSK [31]	2.4251 × 10^−3^	1.04 × 10^−9^	2.4251 × 10^−3^	2.4251 × 10^−3^	
	FDB-TLABC [49]	2.425197 × 10^−2^	3.993488 × 10^−7^	2.425075 × 10^−3^	2.427041 × 10^−3^	-
	ISAO [50]	2.130399 × 10^−2^	6.2532603 × 10^−2^	2.4485452 × 10^−3^	2.49414790 × 10^−1^	-
	I-CPA	6.7743 × 10^−3^	6.1563 × 10^−3^	2.4374 × 10^−3^	3.0363 × 10^−2^	-
	AGDE	4.74 × 10^−3^	9.51 × 10^−3^	2.42 × 10^−3^	5.41 × 10^−3^	-
	SPBO	2.38 × 10^−2^	9.30 × 10^−2^	2.73 × 10^−3^	2.74 × 10^−1^	-
DDM	**CSA**	**2.4250748** × **10^−3^**	**2.0197998** × **10^−10^**	**2.4250748** × **10^−3^**	**2.4250758** × **10^−3^**	**3.8689854** × **10^−12^**
	STF	2.425076 × 10^−3^	1.27 × 10^−8^	2.425075 × 10^−3^	2.425202 × 10^−3^	-
TDM	**CSA**	**2.4328283** × **10^−3^**	**1.7288286** × **10^−5^**	**2.4250749** × **10^−3^**	**2.4833981** × **10^−3^**	**5.7588117** × **10^−6^**
	STF	2.425075 × 10^−3^	1.16 × 10^−10^	2.425075 × 10^−3^	2.425075 × 10^−3^	-

**Table 11:** Values of the computing time for PV device, models and number of iterations.

Models	PV		No. of Iterations				
			1000	1250	1500	1750	2000
SDM	RTC	Computing time [s]	1.190899	1.226915	1.045819	1.45023	1.54682
	aSi:H		0.951085	1.118146	1.346422	1.371934	1.837038
	PWP201		0.814789	0.90668	1.154965	1.581844	1.596234
DDM	RTC		0.981655	1.118023	1.398937	2.029755	2.351117
	aSi:H		1.067067	1.303279	1.534032	1.612922	2.038701
	PWP201		1.06508	1.22953	1.495073	1.583673	2.064425
TDM	RTC		1.15624	1.619865	1.630825	2.34449	2.347905
	aSi:H		1.446611	1.561344	1.806098	2.186155	2.926966
	PWP201		1.551063	1.273363	1.831448	1.887859	2.422485

## Data Availability

No new data were created or analyzed in this study. Data sharing is not applicable to this article.

## Supplementary Materials

The following supporting information can be downloaded at: https://www.mdpi.com/article/10.3390/biomimetics11100706/s1, Table S1. Statistical metrics of RTC Photovoltaic Cell 20 repeated runs with mean, standard deviation, minimum, maximum, and interquartile range for extracted parameters using SDM, DDM, TDM model in the case of 250 Search Agents and 2000 Iterations. Table S2. CSA algorithm-based computed current and errors at the (I, V) points of an RTC Photovoltaic Cell for 250 Search Agents. Table S3. Statistical metrics of aSi:H Photovoltaic Cell 20 repeated runs with mean, standard deviation, minimum, maximum, and interquartile range for extracted parameters using SDM, DDM, TDM model in the case of 250 Search Agents and 1750 Iterations. Table S4. CSA algorithm-based computed current and errors at the (I, V) points of an aSi:H amorphous silicon photovoltaic cell for 250 Search Agents. Table S5. Statistical metrics of PWP201 Photovoltaic Module 20 repeated runs with mean, standard deviation, minimum, maximum, and interquartile range for extracted parameters using SDM, DDM, TDM model in the case of 250 Search Agents and 1750 Iterations. Table S6. CSA algorithm-based computed current and errors at the (I, V) points of an PWP201 Photovoltaic Module for 250 Search Agents.

## Abbreviations

The following abbreviations are used in this manuscript:

BWOA	Black Widow Optimization Algorithm
ECO-Secant	Educational Competition Optimizer-Secant
HSO	Holistic Swarm Optimization
ICSE	Improved Shuffled Complex Evolution
SFOA	Starfish Optimization Algorithm
BMOA	Barnacle Mating Optimizer Algorithm
WDOWOAPSO	Wind-Driven Optimization/Whale Optimization Algorithm/Particle Swarm Optimization
GCPSO	Guaranteed Convergence Particle Swarm Optimization
STF	Special Trans Function
CSA	Chameleon Swarm Algorithm
OBEDO	Opposition-Based Exponential Distribution Optimizer
*μ*AFCSO	Micro Adaptive Fuzzy Cuckoo Search Optimization
FDB-TLABC	Fitness-Distance Balance-Teaching-Learning-Based Artificial Bee Colony
GSK	Gaining-Sharing Knowledge-Based Algorithm
