# Supplementary material for: Chameleon Swarm Algorithm Applied to Extract the Parameters of Photovoltaic Cells and Panels
Source: Biomimetics (Basel). 2026 Oct 3;11(10):706. doi: 10.3390/biomimetics11100706 (PMC13702252; doi:10.3390/biomimetics11100706)
Supplement: Supplementary file 1 [file biomimetics-11-00706-s001.zip › biomimetics-4557920-supplementary.pdf]

Supplementary files

Table S1. Statistical metrics of RTC Photovoltaic Cell 20 repeated runs with mean, standard deviation, minimum, maximum, and interquartile range for extracted parameters using SDM, DDM, TDM model in the case of 250 Search Agents and 2000 Iterations

**Model**	**Iterations**	**Parameters**	**Mean**	**Std_Dev**	**Min**	**Max**	**IQR**	**CV**
SDM	2000	I_{ph}	7.6077553E-01	2.3160454E-10	7.6077553E-01	7.6077553E-01	4.2620640E-10	3.0443E-08
		I_{o1}	3.2302081E-07	2.4327582E-14	3.2302076E-07	3.2302084E-07	3.8624673E-14	7.5313E-06
		n_1	1.4811836E+00	7.6002729E-09	1.4811836E+00	1.4811836E+00	1.2097073E-08	5.1312E-07
		Rs	3.6377093E-02	3.2706715E-10	3.6377092E-02	3.6377093E-02	4.4450450E-10	8.9910E-07
		Rsh	5.3718524E+01	2.8963019E-06	5.3718518E+01	5.3718531E+01	3.6365412E-06	5.3916E-06
DDM	2000	Iph	7.6075073E-01	7.3649317E-05	7.6047160E-01	7.6080135E-01	6.1862401E-05	9.6811E-03
		Io1	2.7468891E-07	7.5528083E-08	5.1410953E-08	4.7031286E-07	6.3540000E-08	2.7496E+01
		n1	1.4766333E+00	2.9998017E-02	1.4505100E+00	1.5749544E+00	2.2176282E-02	2.0315E+00
		Isd2	9.7678546E-07	1.1053526E-06	2.0768844E-12	3.5661149E-06	1.9381286E-06	1.1316E+02
		n2	1.9148321E+00	5.4980250E-01	1.0000000E+00	2.4999232E+00	9.8536263E-01	2.8713E+01
		Rs	3.6666989E-02	3.9962784E-04	3.6238965E-02	3.8179324E-02	2.8723778E-04	1.0899E+00
		Rsh	5.5399773E+01	1.9071860E+00	5.1471534E+01	5.8957415E+01	3.2079465E+00	3.4426E+00
TDM	2000	Iph	7.6076875E-01	3.4153610E-05	7.6069378E-01	7.6083526E-01	3.5065989E-05	4.4894E-03
		Io1	2.5817042E-07	1.1557813E-07	8.8892451E-09	5.5667234E-07	5.1754840E-08	4.4768E+01
		n1	1.4959528E+00	1.0278938E-01	1.4151466E+00	1.9150753E+00	2.9768056E-02	6.8712E+00
		Io2	6.0399079E-07	6.6631026E-07	1.0000000E-12	2.3423220E-06	9.7808174E-07	1.1032E+02
		n2	2.0818243E+00	5.0321204E-01	1.0122583E+00	2.4996718E+00	9.8484381E-01	2.4172E+01
		Io3	7.0045427E-07	8.0869623E-07	1.0000000E-12	2.8476886E-06	1.0652653E-06	1.1545E+02
		n3	2.0604050E+00	4.8283747E-01	1.1320925E+00	2.4998074E+00	8.7563648E-01	2.3434E+01
		Rs	3.6656927E-02	1.5018403E-04	3.6358871E-02	3.6881501E-02	1.9432968E-04	4.0970E-01
		Rsh	5.5602964E+01	1.2322294E+00	5.3844920E+01	5.8243755E+01	1.8206128E+00	2.2161E+00

Table S2. CSA algorithm-based computed current and errors at the (I, V) points of an RTC Photovoltaic Cell for 250 Search Agents

Measured RTC data		SDM-CSA	DDM-CSA	TDM-CSA	Error Values		
V [V]	I_c [A]	I_c [A]	I_c [A]	I_c [A]	SDM	DDM	TDM
−0.2057	0.7640	0.764087704	0.763852318	0.763943526	-8.7703999E-05	1.4768242E-04	5.6473878E-05
−0.1291	0.7620	0.762663086	0.762527516	0.762583282	-6.6308627E-04	-5.2751615E-04	-5.8328216E-04
−0.0588	0.7605	0.761355307	0.761310400	0.761333997	-8.5530717E-04	-8.1040036E-04	-8.3399711E-04
0.0057	0.7605	0.760153991	0.760190047	0.760184942	3.4600905E-04	3.0995282E-04	3.1505820E-04
0.0646	0.7600	0.759055209	0.759160352	0.759130753	9.4479125E-04	8.3964757E-04	8.6924738E-04
0.1185	0.7590	0.758042345	0.758201454	0.758152616	9.5765496E-04	7.9854598E-04	8.4738433E-04
0.1678	0.7570	0.757091654	0.757284222	0.757223088	-9.1653729E-05	-2.8422183E-04	-2.2308790E-04
0.2132	0.7570	0.756141365	0.756340072	0.756275616	8.5863546E-04	6.5992796E-04	7.2438365E-04
0.2545	0.7555	0.755086872	0.755257901	0.755200927	4.1312754E-04	2.4209883E-04	2.9907308E-04
0.2924	0.7540	0.753663878	0.753770833	0.753732569	3.3612203E-04	2.2916712E-04	2.6743100E-04
0.3269	0.7505	0.751390966	0.751404144	0.751393299	-8.9096630E-04	-9.0414369E-04	-8.9329873E-04
0.3585	0.7465	0.747353851	0.747260330	0.747279991	-8.5385127E-04	-7.6032957E-04	-7.7999124E-04
0.3873	0.7385	0.740117222	0.739933327	0.739977399	-1.6172219E-03	-1.4333266E-03	-1.4773988E-03
0.4137	0.7280	0.727382225	0.727154592	0.727207951	6.1777489E-04	8.4540758E-04	7.9204884E-04
0.4373	0.7065	0.706972652	0.706770428	0.706812102	-4.7265159E-04	-2.7042797E-04	-3.1210180E-04
0.4590	0.6755	0.675280152	0.675166194	0.675178558	2.1984823E-04	3.3380643E-04	3.2144237E-04
0.4784	0.6320	0.630758273	0.630760412	0.630738151	1.2417273E-03	1.2395877E-03	1.2618487E-03
0.4960	0.5730	0.571928359	0.572030712	0.571982551	1.0716414E-03	9.6928809E-04	1.0174488E-03
0.5119	0.4990	0.499607019	0.499759031	0.499703662	-6.0701886E-04	-7.5903063E-04	-7.0366151E-04
0.5265	0.4130	0.413648792	0.413778445	0.413739897	-6.4879223E-04	-7.7844479E-04	-7.3989716E-04
0.5398	0.3165	0.317510109	0.317565173	0.317558691	-1.0101095E-03	-1.0651731E-03	-1.0586910E-03
0.5521	0.2120	0.212154939	0.212107879	0.212138919	-1.5493887E-04	-1.0787878E-04	-1.3891910E-04
0.5633	0.1035	0.102251311	0.102121350	0.102178607	1.2486885E-03	1.3786497E-03	1.3213928E-03
0.5736	−0.0100	-0.008717542	-0.008828634	-0.008784421	-1.2824582E-03	-1.1713661E-03	-1.2155793E-03
0.5833	−0.1230	-0.125507413	-0.125558696	-0.125546824	2.5074126E-03	2.5586965E-03	2.5468241E-03
0.5900	−0.2100	-0.208472326	-0.208324856	-0.208391646	-1.5276740E-03	-1.6751442E-03	-1.6083545E-03

Table S3. Statistical metrics of aSi:H Photovoltaic Cell 20 repeated runs with mean, standard deviation, minimum, maximum, and interquartile range for extracted parameters using SDM, DDM, TDM model in the case of 250 Search Agents and 1750 Iterations

Model	Iterations	Parameters	Mean	Std_Dev	Min	Max	IQR	CV
SDM	1750	I_{ph}	1.1344722E-02	1.8466637E-12	1.1344722E-02	1.1344722E-02	1.6769138E-12	1.6278E-08
		I_{o1}	7.3493726E-07	7.5785638E-15	7.3493725E-07	7.3493728E-07	7.6284315E-15	1.0312E-06
		n_1	3.3684180E+00	3.7184412E-09	3.3684180E+00	3.3684180E+00	3.8013890E-09	1.1039E-07
		Rs	5.2021653E-14	4.8010436E-14	6.4308380E-15	1.5720136E-13	7.1203160E-14	9.2289E+01
		Rsh	5.2315342E+02	1.5346776E-06	5.2315341E+02	5.2315342E+02	1.9911172E-06	2.9335E-07
DDM	1750	Iph	1.1327377E-02	7.5147888E-06	1.1320118E-02	1.1345839E-02	7.1062404E-06	6.6342E-02
		Io1	2.1650056E-06	2.3083537E-06	1.6992352E-08	5.5421610E-06	4.4875750E-06	1.0662E+02
		n1	3.8559092E+00	1.0404529E+00	2.6073076E+00	4.9999999E+00	2.0527474E+00	2.6983E+01
		Isd2	1.8094801E-06	2.0448795E-06	5.8021145E-11	4.8471034E-06	4.2541979E-06	1.1301E+02
		n2	3.7090403E+00	1.0079285E+00	2.0697986E+00	5.0000000E+00	2.0744870E+00	2.7175E+01
		Rs	7.1061196E-02	1.3148789E-01	4.0377247E-04	4.9787087E-01	6.5262387E-02	1.8503E+02
		Rsh	5.7985788E+02	2.3423869E+01	5.2595755E+02	6.0380376E+02	2.3355550E+01	4.0396E+00
TDM	1750	Iph	1.1329875E-02	5.2364695E-06	1.1322516E-02	1.1342073E-02	7.7997363E-06	4.6218E-02
		Io1	1.6618790E-06	2.1600449E-06	1.0000000E-12	5.2512676E-06	4.0978095E-06	1.2998E+02
		n1	3.9491753E+00	1.0775568E+00	2.5273181E+00	5.0000000E+00	2.0635977E+00	2.7286E+01
		Io2	1.0075140E-06	1.7769093E-06	1.0000000E-12	5.5074246E-06	8.4846597E-07	1.7637E+02
		n2	4.0250655E+00	9.9132482E-01	2.6752921E+00	5.0000000E+00	2.0313017E+00	2.4629E+01
		Io3	2.1546744E-06	2.3090135E-06	1.7215070E-08	5.5989096E-06	4.5234764E-06	1.0716E+02
		n3	4.1200871E+00	1.0845677E+00	2.5947065E+00	5.0000000E+00	2.1344214E+00	2.6324E+01
		Rs	2.5533167E-01	2.3496866E-01	3.7806584E-05	5.0000000E-01	4.9663388E-01	9.2025E+01
		Rsh	5.8933842E+02	1.2465236E+01	5.5016298E+02	6.0629950E+02	1.7393457E+01	2.1151E+00

Table S4. CSA algorithm-based computed current and errors at the (I, V) points of an aSi:H amorphous silicon photovoltaic cell for 250 Search Agents

Measured aSi:H data		SDM-CSA	DDM-CSA	TDM-CSA	Error Values		
V [V]	I_c [A]	I_c [A]	I_c [A]	I_c [A]	SDM	DDM	TDM
-0.01	0.011283	0.011363917	0.011339331	0.011339178	-8.0817076E-05	-5.6231349E-05	-5.6077662E-05
0.00848	0.011266	0.011328437	0.011307738	0.011307589	-6.2836972E-05	-4.2138459E-05	-4.1988860E-05
0.026955	0.011243	0.011292929	0.011276027	0.011275882	-5.0429382E-05	-3.3527098E-05	-3.3381825E-05
0.045435	0.011224	0.011257366	0.011244151	0.011244010	-3.3766257E-05	-2.0550674E-05	-2.0409977E-05
0.063915	0.011202	0.011221746	0.011212099	0.011211963	-1.9346268E-05	-9.6988566E-06	-9.5630338E-06
0.08239	0.011193	0.011186066	0.011179840	0.011179709	7.0344534E-06	1.3260489E-05	1.3391130E-05
0.10087	0.011155	0.011150288	0.011147358	0.011147233	4.4119755E-06	7.3422054E-06	7.4672286E-06
0.11935	0.01111	0.011114403	0.011114604	0.011114485	-4.0026018E-06	-4.2036051E-06	-4.0846365E-06
0.137825	0.011099	0.011078393	0.011081506	0.011081393	2.0606641E-05	1.7494095E-05	1.7606600E-05
0.156305	0.011078	0.011042209	0.011048031	0.011047926	3.5990985E-05	3.0168882E-05	3.0274369E-05
0.174785	0.01104	0.011005820	0.011014134	0.011014036	3.3880073E-05	2.5566228E-05	2.5664062E-05
0.19326	0.011007	0.010969187	0.010979729	0.010979639	3.7812688E-05	2.7271217E-05	2.7360746E-05
0.21175	0.010982	0.010932211	0.010944691	0.010944610	4.9389080E-05	3.6909422E-05	3.6989917E-05
0.2302	0.010941	0.010894927	0.010909045	0.010908974	4.6373126E-05	3.2255420E-05	3.2326060E-05
0.2487	0.010896	0.010857060	0.010872487	0.010872427	3.9139568E-05	2.3713077E-05	2.3772934E-05
0.26715	0.010861	0.010818702	0.010835059	0.010835011	4.2297907E-05	2.5940969E-05	2.5989132E-05
0.28565	0.010838	0.010779502	0.010796386	0.010796351	5.7997914E-05	4.1113912E-05	4.1149337E-05
0.30415	0.010777	0.010739387	0.010756418	0.010756397	3.7912529E-05	2.0881651E-05	2.0903148E-05
0.3226	0.010724	0.010698254	0.010714985	0.010714979	2.5246497E-05	8.5149129E-06	8.5213755E-06
0.3411	0.01069	0.010655608	0.010671557	0.010671566	3.3992011E-05	1.8043315E-05	1.8033556E-05
0.35955	0.010624	0.010611349	0.010626085	0.010626113	1.2350668E-05	-2.3854638E-06	-2.4126827E-06
0.37805	0.010596	0.010564825	0.010577820	0.010577866	3.1074989E-05	1.8079663E-05	1.8033867E-05
0.3965	0.010523	0.010515778	0.010526630	0.010526695	6.8224840E-06	-4.0297668E-06	-4.0953056E-06
0.415	0.010467	0.010463309	0.010471539	0.010471625	3.7913922E-06	-4.4392714E-06	-4.5254946E-06
0.4335	0.010407	0.010406763	0.010411967	0.010412074	5.3731449E-07	-4.6666128E-06	-4.7742322E-06

0.45195	0.010327	0.010345342	0.010347212	0.010347342	-1.8142362E-05	-2.0012434E-05	-2.0141812E-05
0.47045	0.010258	0.010277515	0.010275754	0.010275905	-2.0014539E-05	-1.8254230E-05	-1.8405235E-05
0.4889	0.010173	0.010202165	0.010196657	0.010196829	-2.9665400E-05	-2.4157135E-05	-2.4328959E-05
0.5074	0.01006	0.010117047	0.010107831	0.010108022	-5.7247150E-05	-4.8031100E-05	-4.8222262E-05
0.52585	0.009967	0.010020351	0.010007543	0.010007751	-5.3350974E-05	-4.0543371E-05	-4.0750908E-05
0.54435	0.009853	0.009908734	0.009892734	0.009892954	-5.5333745E-05	-3.9334269E-05	-3.9554146E-05
0.56285	0.009702	0.009778943	0.009760465	0.009760692	-7.6842551E-05	-5.8365313E-05	-5.8591949E-05
0.5813	0.009567	0.009627092	0.009606899	0.009607125	-6.0192443E-05	-3.9999331E-05	-4.0224684E-05
0.5998	0.009384	0.009447010	0.009426351	0.009426566	-6.3210310E-05	-4.2551422E-05	-4.2765837E-05
0.61825	0.009201	0.009233070	0.009213163	0.009213353	-3.1970204E-05	-1.2062581E-05	-1.2253420E-05
0.63675	0.008975	0.008975913	0.008958317	0.008958470	-1.1131315E-06	1.6482853E-05	1.6330239E-05
0.6552	0.008685	0.008666815	0.008653248	0.008653346	1.8484926E-05	3.2052099E-05	3.1953751E-05
0.6737	0.008338	0.008291534	0.008283478	0.008283504	4.6065583E-05	5.4122379E-05	5.4095896E-05
0.69215	0.007911	0.007836599	0.007835180	0.007835119	7.4400907E-05	7.5819800E-05	7.5881185E-05
0.71065	0.007373	0.007280284	0.007286108	0.007285947	9.2215524E-05	8.6392035E-05	8.6552792E-05
0.72915	0.006665	0.006599810	0.006613078	0.006612818	6.5189820E-05	5.1922447E-05	5.2181578E-05
0.7476	0.005787	0.005768078	0.005787271	0.005786934	1.8922091E-05	-2.7088551E-07	6.6496782E-08
0.7661	0.004701	0.004744029	0.004765832	0.004765470	-4.2629324E-05	-6.3445962E-05	-6.4069757E-05
0.78455	0.003403	0.003488115	0.003506000	0.003505717	-8.4714740E-05	-1.0202119E-04	-1.0231707E-04
0.80305	0.001877	0.001937511	0.001940333	0.001940310	-6.0610733E-05	-6.3663957E-05	-6.3410487E-05
0.8215	9.58E-05	0.000031502	0.000003456	0.000003993	6.4294997E-05	9.0736054E-05	9.1804709E-05

Table S5. Statistical metrics of PWP201 Photovoltaic Module 20 repeated runs with mean, standard deviation, minimum, maximum, and interquartile range for extracted parameters using SDM, DDM, TDM model in the case of 250 Search Agents and 1750 Iterations

Model	Iterations	Parameters	Mean	Std_Dev	Min	Max	IQR	CV
SDM	1750	I_{ph}	1.0305143E+00	7.0088929E-10	1.0305143E+00	1.0305143E+00	9.4023012E-10	6.8014E-08
		I_{o1}	3.4822629E-06	2.4525875E-13	3.4822623E-06	3.4822633E-06	2.9892493E-13	7.0431E-06
		n_1	1.351190	7.47969E-09	1.351190	1.351190	9.024822E-09	5.5356E-07
		Rs	1.2012710E+00	7.3089351E-09	1.2012710E+00	1.2012710E+00	7.1059549E-09	6.0843E-07
		Rsh	9.8198227E+02	9.7263047E-05	9.8198203E+02	9.8198245E+02	1.0131920E-04	9.9048E-06
DDM	1750	Iph	1.0305344E+00	1.0178076E-04	1.0304223E+00	1.0309228E+00	2.3307833E-09	9.8765E-03
		Io1	2.0190852E-06	1.5898238E-06	1.0000000E-12	3.4822631E-06	3.4817288E-06	7.8740E+01
		n1	1.448682	2.06756E-01	1.351115	1.997258	3.306152E-02	1.4272E+01
		Isd2	1.4594804E-06	1.5847553E-06	1.0000000E-12	3.4822629E-06	3.4817278E-06	1.0858E+02
		n2	1.531001	2.35249E-01	1.343547	1.982489	3.889214E-01	1.5366E+01
		Rs	1.2013816E+00	7.2618565E-04	1.2006672E+00	1.2043427E+00	1.7681609E-08	6.0446E-02
		Rsh	9.7936620E+02	1.3746881E+01	9.3103645E+02	9.9573978E+02	3.2564287E-04	1.4037E+00
TDM	1750	Iph	1.0305748E+00	1.5850002E-04	1.0303259E+00	1.0308879E+00	1.4239143E-04	1.5380E-02
		Io1	1.2948317E-06	1.5572951E-06	1.0000000E-12	3.5091073E-06	3.3794191E-06	1.2027E+02
		n1	1.550950	2.79868E-01	1.229329	2.000000	5.025587E-01	1.8045E+01
		Io2	1.6738260E-06	1.5402315E-06	1.0000000E-12	3.6131059E-06	3.4417435E-06	9.2019E+01
		n2	1.442303	1.64190E-01	1.316652	1.915342	1.428964E-01	1.1384E+01
		Io3	6.6198934E-07	1.2660352E-06	1.0000000E-12	3.4822619E-06	3.6442970E-07	1.9125E+02
		n3	1.630263	2.71602E-01	1.176927	1.999976	5.987020E-01	1.6660E+01
		Rs	1.2015820E+00	3.2110333E-03	1.1944507E+00	1.2108706E+00	9.5625981E-04	2.6723E-01
		Rsh	9.7637660E+02	1.8181769E+01	9.3350441E+02	9.9972200E+02	7.8083083E+00	1.8622E+00

Table S6. CSA algorithm-based computed current and errors at the (I, V) points of an PWP201 Photovoltaic Module for 250 Search Agents

Measured RTC data		SDM-CSA	DDM-CSA	TDM-CSA	Error Values		
V [V]	I_c [A]	I_c [A]	I_c [A]	I_c [A]	SDM	DDM	TDM
0.1248	1.0315	1.029119161	1.029119162	1.029131257	2.3808395E-03	2.3808381E-03	2.3687426E-03
1.8093	1.0300	1.027381073	1.027381074	1.027388362	2.6189274E-03	2.6189265E-03	2.6116379E-03
3.3511	1.0260	1.025741797	1.025741797	1.025744755	2.5820349E-04	2.5820289E-04	2.5524520E-04
4.7622	1.0220	1.024107155	1.024107155	1.024106281	-2.1071545E-03	-2.1071548E-03	-2.1062813E-03
6.0538	1.0180	1.022291804	1.022291804	1.022287686	-4.2918045E-03	-4.2918044E-03	-4.2876857E-03
7.2364	1.0155	1.019930681	1.019930681	1.019924052	-4.4306809E-03	-4.4306806E-03	-4.4240518E-03
8.3189	1.0140	1.016363106	1.016363105	1.016354924	-2.3631058E-03	-2.3631052E-03	-2.3549237E-03
9.3097	1.0100	1.010496151	1.010496151	1.010487622	-4.9615147E-04	-4.9615075E-04	-4.8762242E-04
10.2163	1.0035	1.00062897	1.000628969	1.000621471	2.8710301E-03	2.8710309E-03	2.8785293E-03
11.0449	0.9880	0.984548379	0.984548378	0.984543257	3.4516214E-03	3.4516222E-03	3.4567426E-03
11.8018	0.9630	0.959521676	0.959521675	0.959519932	3.4783239E-03	3.4783246E-03	3.4800684E-03
12.4929	0.9255	0.922838818	0.922838817	0.922840775	2.6611821E-03	2.6611825E-03	2.6592252E-03
13.1231	0.8725	0.872599663	0.872599662	0.872604813	-9.9662592E-05	-9.9662381E-05	-1.0481273E-04
13.6983	0.8075	0.807274263	0.807274263	0.807281272	2.2573660E-04	2.2573658E-04	2.1872804E-04
14.2221	0.7265	0.728336478	0.728336478	0.728343731	-1.8364777E-03	-1.8364779E-03	-1.8437314E-03
14.6995	0.6345	0.637138	0.637138	0.637143882	-2.6379998E-03	-2.6380001E-03	-2.6438816E-03
15.1346	0.5345	0.536213063	0.536213063	0.536216352	-1.7130630E-03	-1.7130634E-03	-1.7163515E-03
15.5311	0.4275	0.429511325	0.429511325	0.429511679	-2.0113248E-03	-2.0113252E-03	-2.0116789E-03
15.8929	0.3185	0.318774483	0.318774483	0.318771952	-2.7448278E-04	-2.7448307E-04	-2.7195228E-04
16.2229	0.2085	0.207389507	0.207389507	0.207384912	1.1104933E-03	1.1104931E-03	1.1150878E-03
16.5241	0.1010	0.096167172	0.096167172	0.096161377	4.8328282E-03	4.8328281E-03	4.8386233E-03
16.7987	−0.0080	-0.008325386	-0.008325386	-0.008329888	3.2538615E-04	3.2538613E-04	3.2988804E-04
17.0499	−0.1110	-0.110936483	-0.110936483	-0.11093878	-6.3517172E-05	-6.3517114E-05	-6.1219534E-05
17.2793	−0.2090	-0.209247266	-0.209247266	-0.209246076	2.4726613E-04	2.4726625E-04	2.4607639E-04
17.4885	−0.3030	-0.300863587	-0.300863587	-0.300857249	-2.1364127E-03	-2.1364126E-03	-2.1427512E-03
